# Integrated metabolomic and transcriptomic analysis identifies adipogenic differentiation of mesenchymal stem cells as a driver of chemoresistance in acute myeloid leukemia

**DOI:** 10.1186/s13046-025-03550-0

**Published:** 2025-10-17

**Authors:** Zhipeng Pan, Rong Hu, Dandan Li, Siwen Deng, Haishan Yi, Zhengwei Duan, Lixia Kang, Ling Chen, Mengyao Wang, Yue Duan, Xiaofan Jia, Pengfei Guo, Yang Chen

**Affiliations:** 1 Department of Laboratory Medicine, Fujian Medical University, Fuzhou, 350122 China; 2 Key Laboratory of Clinical Laboratory Technology for Precision Medicine (Fujian Medical University), Fujian Province University, Fujian Medical University, Fuzhou, 350122 China; 3 Department of Clinical Laboratory, Sir Run Run Shaw Hospital, Zhejiang University School of Medicine, Hangzhou, 310016 China; 4 Department of Clinical Laboratory, Fujian Medical University Union Hospital, Fuzhou, 350001 China; 5 Fuzhou Second General Hospital, Fuzhou, 350007 China; 6 Department of Electronic Science, Fujian Provincial Key Laboratory of Plasma and Magnetic Resonance, Xiamen University, Xiamen, 361005 China

**Keywords:** Co-culture, Acute myeloid leukemia, Mesenchymal stem cells, Adipogenic differentiation, Chemosensitivity

## Abstract

**Background:**

Acute myeloid leukemia (AML) remains a challenging hematological malignancy, with chemoresistance contributing significantly to treatment failure and relapse. The bone marrow microenvironment, particularly mesenchymal stem cells (MSCs), plays a critical role in AML cell survival and drug resistance. Although previous studies have extensively explored the MSCs differentiation, the regulatory role of the adipogenically differentiated MSCs on AML cells during co-culture remains unclear.

**Methods:**

An indirect co-culture model was established to evaluate the impact of MSCs on the drug sensitivity of AML cells. Based on the comparable chemosensitivity trends observed among THP-1, U937, and HL-60 cells, THP-1 were selected for subsequent experiments due to their stable growth characteristics and well-established utilization. Metabolic alterations between co-cultured and monocultured THP-1 were profiled using nuclear magnetic resonance spectroscopy. Concurrently, RNA sequencing was conducted to identify differentially expressed genes and enriched signaling pathways between co-cultured and monocultured THP-1. To validate the pathway alterations identified by transcriptomic analysis, the Akt inhibitor MK-2206 was applied, and its effects were evaluated by western blotting and cell viability assays.

**Results:**

The results demonstrated that AML cells co-cultured with adipogenic MSCs were less sensitive to daunorubicin and cytarabine in both in vitro and in vivo. Subsequent metabolomics analysis revealed significant alternative metabolic processes in AML cells following co-culture, specifically in the glycolysis, glutamine metabolism and lipid metabolism. Further transcriptomic profiling identified key differentially expressed genes and signaling pathways, with PI3K/Akt signaling pathway activation emerging as a contributor to the reduced chemotherapy sensitivity. Furthermore, elevated levels of IL-6 in the co-culture system suggested a role for cytokine-mediated signaling in promoting a protective microenvironment.

**Conclusions:**

This work demonstrates that the adipogenically differentiated MSCs enhance the survival and chemoresistance of AML cells by modulating metabolic and signaling pathways. It provides integrated insights into the microenvironment-driven mechanisms of AML drug resistance and presents potential therapeutic targets to enhance treatment efficacy.

**Supplementary Information:**

The online version contains supplementary material available at 10.1186/s13046-025-03550-0.

## Background

Acute myeloid leukemia (AML) is a malignant bone marrow disease characterized by clonal proliferation and arrested differentiation of bone marrow progenitor cells [1]. Chemotherapy is the primary treatment and achieves complete response rates of 75%-80%. However, more than 50% of patients eventually relapse after treatment [2]. This relapse is closely linked to reduced chemosensitivity in AML cells, which is driven by multiple resistance mechanisms. One key mechanism is the increased expression of drug efflux pumps like P-glycoprotein (P-gp), which lowers intracellular drug concentrations [3]. Drug efflux pumps are often regulated by specific signaling pathways. For example, the NF-κB signaling pathway participates in the regulation of P-gp expression [4]. Inhibition of NF-κB signaling pathway decreases P-gp levels and consequently increases sensitivity to doxorubicin in resistant AML cells. Moreover, mutations in genes such as *FLT3* or *TP53*, along with alterations in downstream signaling pathways, contribute to the survival and proliferation of residual leukemic cells during treatment [5]. Identifying these resistance mechanisms has provided new insights into the complexities of drug resistance in AML and highlights the need for further research into other potential resistance factors.

Emerging evidence suggests that the bone marrow microenvironment (BMM) significantly contributes to chemoresistance in AML, alongside well-known cellular and genetic mechanisms [6]. Initially described in 1978, the BMM provides structural support and actively regulates hematopoiesis [7, 8]. As essential components of the BMM, the mesenchymal stem cells (MSCs) are multipotent cells capable of differentiating into osteoblasts, adipocytes, and other mesenchymal lineages. Beyond their physiological roles, the MSCs support leukemia cell survival by secreting growth factors, extracellular matrix components, and other protective molecules that enhance leukemia cell resistance to chemotherapy [9–11]. One mechanism by which the MSCs contribute to drug resistance is through asparagine metabolism. In a category of acute leukemia known as acute lymphoblastic leukemia, the cancer cells have limited aspartate synthesis, making aspartate-targeting enzymes crucial for therapy. However, MSCs protect ALL cells from L-asparaginase by using glutamine supplied by leukemic blasts to produce and export asparagine, thereby supporting leukemic cell survival [12].

In AML, the MSCs transfer functional mitochondria to leukemic cells, thereby enhancing mitochondrial energy metabolism and promoting cell survival under chemotherapeutic stress [13]. A specific subset of the MSCs, nestin-positive MSCs, further contributes to AML chemoresistance by boosting oxidative phosphorylation and supplying glutathione, thereby strengthening energy metabolism and antioxidant defense mechanisms in AML cells [14]. These studies indicate that the MSCs alter energy metabolism, contributing to leukemia cell survival during chemotherapy. Furthermore, a variety of cytokines are another key way to enhance the chemotherapy resistance of AML cells. On the one hand, cytokines indirectly promote leukemic cell survival by acting through MSCs to modulate the microenvironment. In a recent finding, co-culture with the MSCs has been shown to induce a stem cell-like phenotype in leukemic cells, increasing their resistance to chemotherapy [15]. This effect is mediated by interleukin-4 signaling, which upregulates vascular cell adhesion molecule-1 in the MSCs. As a result, AML cells exhibit enhanced adhesion to the stromal environment, further promoting drug resistance. On the other hand, cytokines secreted by MSCs directly regulate AML cells by activating signaling pathways. For example, the AML-MSCs secreted elevated levels of interleukin-6 (IL-6), subsequently activating the JAK2/STAT3 signaling pathway within AML cells [16]. This promoted the expression of epithelial–mesenchymal transition markers, particularly in drug-resistant AML cells, thus contributing to chemotherapy resistance. Additionally, the MSCs also regulate AML progression through the release of exosomes, which facilitate leukemic cell proliferation, invasion, and chemoresistance [17]. These findings underscore the critical role of the MSCs in modulating AML cell behavior. Concurrently, the AML cells conversely regulate the MSCs by reshaping of the bone marrow microenvironment [18, 19]. The regulatory mechanism may be initiated through the induction of metabolic changes or the modulation of the differentiation potential of the MSCs [20, 21], thereby fostering the survival and proliferation of the leukemic cells. These interactions indicated the significance and necessity of co-culturing AML cells with the MSCs within a shared environment for AML-related research. Although previous studies have extensively explored the MSCs differentiation [22, 23], the regulatory role of the adipogenically differentiated MSCs on AML cells during co-culture remains unclear.

In this study, distinct differences in the adipogenic differentiation process of the MSCs were observed. The MSCs exhibiting different levels of adipogenic differentiation were associated with varying prognoses in AML patients. Besides, the AML cells were co-cultured with adipogenic MSCs from AML patients, to investigate how these MSCs contributed to AML cell survival and protection, with a specific focus on elucidating mechanisms behind enhanced resistance to daunorubicin (DNR) and cytarabine (Ara-C). Transcriptomic and metabolomic analyses were conducted to comprehensively characterize the regulatory mechanisms responsible for drug resistance development in this co-culture system. Information about the microenvironment action mechanisms of drug resistance in AML may contribute to develop novel therapeutic strategies for overcoming chemoresistance in AML patients.

## Methods

### Isolation and culture of the MSCs

Human MSCs from bone marrow samples were obtained from AML patients in the Department of Hematology, Fujian Medical University Union Hospital. This study was carried out in accordance with the provisions of Helsinki Declaration on human experiments and approved by the Ethics Committee of Fujian Medical University Union Hospital. All patients obtained informed consent prior to participating. Bone marrow mononuclear cells were isolated using Ficoll-Hypaque density centrifugation and subsequently cultured in low glucose Dulbecco’s modified Eagle medium (L-DMEM, SH30021, Hyclone, USA), which contained 20% fetal bovine serum (FBS, A5256701, Gibco, USA) and 1% penicillin-streptomycin (PS, 15140-122, Gibco, USA). The cultures were in a humidified 5% CO_2_ atmosphere at 37℃ for a period of 7 days. The medium was then replaced to remove the suspended non-MSCs cells, after which the adherent cells (contain MSCs) were continued to be cultivated. Upon achieving a cell density of 90%, the adherent cells were transferred to a culture medium comprising RPMI 1640 (SH30255, Hyclone, USA), with the addition of 10% FBS, 2 mM L-glutamine (25030081, Gibco, USA), and 1% PS. The medium was employed to support the growth of the MSCs, and their morphology was observed under the inverted microscope (TS-100, Nikon, Japan). Subsequently, the proliferation capacity of the isolated MSCs was assessed using a cloning formation experiment. The MSCs cell suspension was harvested and diluted to create a gradient, which was then inoculated into the six-well plates (tc-treated, 140675, Thermo Fisher, USA) at a density of 500, 1000, and 2000 cells per well, respectively. After a culture period of 14 days, the cells were fixed with 4% methyl alcohol for 30 min and then incubated with Giemsa stain for 30 min at room temperature. The quantity of colonies formed by the MSCs was observed under the microscope.

### Induce differentiation of the MSCs

After passaging and expansion, the MSCs were seeded at a cell density of 2 × 10^5^ cells/well in 2 mL of complete medium per well in six-well plates. Once the cell fusion degree reached 60%~70%, osteogenic differentiation complete medium (HUXMX-90021, Cyagen Biosciences, USA) was added. Following a 14-days bone formation induction period, the osteogenic differentiation was identified through Alizarin Red S staining (G8550, Solarbio, China). For the adipogenic differentiation, the induction medium consisted of adipogenic medium A and B (HUXMX-90031, Cyagen Biosciences, USA). Once the cell fusion degree reached 100% or above, 2 mL medium A was changed to continue culture. After 3 days of culturing, 2 mL medium B was used to perform a medium change and cultured for 24 h. Following a three-cycle alternation between medium A and B, the culture continued in medium B for a period of 4 days, until the lipid droplets had become sufficiently large. Lastly, the Oil Red O staining (G1262, Solarbio, China) was used to identify the fat formation. Both differentiation induction processes lasted 14 days, with no additional passaging performed during or after this period.

### Detection of the MSCs surface markers by flow cytometry

The MSCs were evenly distributed into 7 flow cytometry tubes with ≥ 2 × 10^5^ cells per tube, and were resuspended in 100 µL of PBS. Each tube was stained with 5 µL of a PE (P-phycoerythrin)-conjugated monoclonal antibody against one of the following surface markers [24–26]: CD34, CD44, CD45, CD73, CD90, CD105 (14-0341-82, 14-0441-82, 14-0451-82, 17-0739-42, 14-0909-82, 17-1057-42, eBiosciences, USA). PE-conjugated mouse IgG isotype control antibodies (31903, eBioscience, USA) were used in parallel to assess non-specific binding. Each marker was analyzed in an independent run. Surface antigen expression was detected by flow cytometry (Accuri C6 Plus, BD Biosciences, USA). The experiment was independently repeated three times.

### Establish co-culture system and cell counting kit-8 method for chemosensitivity detection

Human AML cell lines HL-60, U937 and THP-1 were preserved in our laboratory, and cultured in RPMI 1640 with 10% FBS and 1% PS. Subsequently, these cell lines (HL-60, U937 and THP-1) were cultured separately and inoculated into a six-well plates (tc-treated, 140675, Thermo Fisher, USA) at a concentration of 3.0 × 10^5^ cells/mL. To investigate the effects of MSCs on AML cells, the transwell indirect co-culture system (3450, Corning, USA) was employed with RPMI 1640. AML cells were seeded in the upper chamber of a six-well transwell insert with a pore size of 0.4 μm, while MSCs were cultured in the lower chamber. This setup allows the exchange of soluble factors without direct cell-to-cell contact. After 24 h, AML cells from both the co-culture and monoculture groups were added to medium containing 5 µL 100 mg/mL DNR (ID2260, Solarbio, China) or 10 µM Ara-C (IC0630, Solarbio, China). The cells were then incubated in an incubator set to 37℃ and 5% CO_2_-saturated humidity for a period of 24 h. Finally, following 4 h of addition and incubation with 10 µL of cell counting kit-8 (CCK-8, CK04-01, Dojindo Laboratories, Japan), the absorbances of per well at 450 nm and 630 nm were measured using a microplate reader (Labserv K3 Touch, Thermo Fisher, USA). The cell viability (%) was calculated as:

Viability=[(OD_treated_−OD_blank_)/(OD_control_−OD_blank_)]×100%.

### Murine tumor-forming experiment

The work has been reported consistent with the ARRIVE guidelines 2.0.

Given the comparable trends observed among THP-1, U937, and HL-60 cells in chemosensitivity experiments, the THP-1 was selected for follow-up mouse model studies due to its stable growth characteristics and well-established utilization. The experiment included two groups, the co-culture group and the monoculture group (control group), and each group had 6 animals as the experimental unit. Six animals per group was considered sufficient to detect biologically relevant differences according to the 3Rs principle (replacement, reduction and refinement). The co-culture group received THP-1 and MSCs suspension, while the monoculture group received THP-1 suspension. A total of twelve NOD-SCID mice (4–6 weeks old, 18–22 g, female) were used. The mice were housed in cages under standard conditions for one week. Their health was monitored throughout the experiment and they were excluded if they showed signs of illness, infection, injury or behavioral abnormalities before or during the study. Twelve mice were randomly divided into two groups, and the mice were labeled from 1 to 12. Then a random number generator was used to select six unique numbers between 1 and 12. The mice with these numbers were assigned to the monoculture group, and the remaining six were assigned to the co-culture group. The THP-1 cells co-cultured with the MSCs and those monocultured with THP-1 cells were collected at the logarithmic growth phase. Anaesthesia was induced in all mice by intraperitoneal injection of pentobarbital sodium (1%, 100 mg/kg). The previously collected cell concentration was adjusted to 1 × 10^6^ cells/mL. For tumor formation, the co-culture group was subcutaneously injected into the left axilla with a 100 µL mixture containing THP-1 suspension and MSCs suspension, while the monoculture group received 100 µL of THP-1 suspension at the same site. Subcutaneous implantation was chosen because intravenous and intramedullary injections had failed to achieve stable engraftment of human MSCs, likely due to their xenogeneic nature, larger cell size, and limited retention in the murine circulation and bone marrow cavity. These mice were fed for a period of two weeks prior to the commencement of Ara-C treatment. The Ara-C (75 mg/kg) was administered via intraperitoneal injection on alternate days. Tumor size was monitored daily. After a 14-days of treatment period, mice were euthanized, and subcutaneous tumors were excised. For euthanasia, following induction of anesthesia with pentobarbital sodium and confirmation of the stage of Surgical Anesthesia, euthanasia was performed by cervical dislocation. The dimensions of the tumors were then measured using vernier calipers. Tumor volume and weight were recorded. The collected tumors were analyzed blindly by a two-tailed Student’s *t*-test using GraphPad Prism 8.0 (GraphPad Software, USA).

### Sample preparation and NMR metabolomics analysis

THP-1 from the co-cultured group and monocultured group was collected and added with 0.6 mL ultrapure water, followed by 1.2 mL methanol (4℃) extract for metabolic quenching. After that, 1.2 mL of CHCl_3_ and 1.2 mL of ultrapure water were added, shaken, and mixed evenly. The mixture was centrifuged at 10,000 rpm for 10 min, after which the supernatant was taken. A vacuum dryer (VOS-310 C, EYELA, Japan) was used to dry the supernatant at 40℃ to about 1/2 of the original liquid level. Then the sample was frozen over night at -20℃. The next day, a vacuum freeze dryer (FDU-2200, EYELA, Japan) was used to collect the powder by freeze dry over night. Subsequently, this was followed by the addition of 550 µL of D_2_O to prepare a phosphate buffer containing 0.1% sodium 2,2,3,3-d-(4)-3-(trimethylsilyl) propionate (TSP) (with a ratio of 90 mM K_2_HPO_4_/NaH_2_PO_4_ = 1/4, pH = 7.4). After mixing, the sample was centrifuged at 10,000 rpm and 4℃ for 10 min. The supernatant was transferred to a 5 mm NMR tube for NMR measurement. At the same time, the MSCs-THP-1 co-culture medium was centrifuged at 4℃ and 10,000 rpm for 10 min, and 400 µL supernatant and 200 µL buffer containing 0.05% TSP were mixed evenly. Then the supernatant was placed in a 5 mm NMR tube for NMR measurement. The ^1^H NMR spectra were obtained at 298 K of operating temperature using a 600 MHz Bruker Avance III NMR spectrometer (Bruker, German). The NMR spectra were preprocessed on MestReNova 14.2 (Mestrelab Research, Santiago de Compostela, Spain). Furthermore, the metabolite peaks identified in the ^1^H NMR spectra were assigned using ChenomX NMR Suit software (ChenomX Inc, Canada), and confirmed by published data and the Human Metabolome Database (HMDB, http://www.hmdb.ca), thereby ensuring accuracy and reliability.

The processed NMR data were imported into SIMCA 14.1 (Umetrics, Umea, Sweden) for pattern recognition analysis, including principal component analysis (PCA) and orthogonal partial least squares with discriminant analysis (OPLS-DA). The variable importance projection (VIP) and *p*-value were used to screen differential metabolites. Ultimately, MetaboAnalyst 6.0 (http://www.metaboanalyst.ca) was used to analyze the metabolic pathways.

### Transcriptome sequencing analysis

Total RNA of THP-1 cells was extracted using Trizol in both the co-culture and monoculture groups. The purity and concentration of the RNA were measured using a Nanodrop spectrophotometer (NanoDrop One, Thermo Fisher, USA). Qualified RNA samples were reverse-transcribed to construct the complementary DNA (cDNA) library. The quality of the constructed libraries was assessed to ensure they met the required standards. Next-generation sequencing (NGS) was performed using paired-end sequencing. After sequencing, raw data were filtered to remove low-quality reads. The high-quality reads were then aligned to the reference genome, and gene expression values were quantified using the Salmon tool [27]. Differentially expressed genes (DEGs) were identified using the DESeq2 package in R, with *p* ≤ 0.05 as the threshold for statistical significance. Kyoto Encyclopedia of Genes and Genomes (KEGG) pathway enrichment analysis was performed on the DEGs using the cluster Profiler R package. The *p* ≤ 0.05 was considered statistically significant.

### Western blotting

THP-1 extracts were obtained using RIPA lysis buffer composed of 50 mM Tris-HCl (pH = 7.5), 15 mM NaCl, 1% NP-40, 0.5% deoxycholate, 0.1% sodium dodecyl sulfate, and supplemented with protease inhibitors (1:100 dilution, Halt™ Protease and Phosphatase Inhibitor Cocktail[100X], 78440, Thermo Fisher, USA). For western blotting (WB), 30 µg of total protein was loaded onto an 10% sodium dodecyl sulfate-polyacrylamide gel and subsequently transferred to a nitrocellulose membrane. The membrane was blocked over night at 4℃ in TBST (Tris-buffered saline, containing 0.2% Tween-20, WB-0151, Dingguo, China) supplemented with 5% phospho-blocker (Halt™ Protease and Phosphatase Inhibitor Cocktail[100X], 78440, Thermo Fisher, USA). Subsequently, the membrane was incubated over night at 4℃ with the following primary antibodies: anti-PI3K (1:1,000 dilution, ab191606, Abcam, USA), anti-p-PI3K (1:1,000 dilution, ab278545, Abcam, USA), anti-Akt (1:1,000 dilution, ab8805, Abcam, USA), anti-p-Akt (1:1,000 dilution, ab81283, Abcam, USA), anti-β-actin (1:1,000 dilution, ab8227, Abcam, USA) and anti-GAPDH (1:1,000 dilution, ab181602, Abcam, USA). Following primary antibody incubation, secondary HRP-conjugated antibodies were incubated in 5% (wt/vol) nonfat dried milk in TBST. Visualization was achieved using ChemiDOX^TM^XRS+ Universal Hood II and the Image Lab system (BIO-RAD, California, USA).

### Inhibition of PI3K/Akt signaling pathway

To inhibit the PI3K/Akt signaling pathway, the selective inhibitor MK-2206 (S1078, Selleck Chemicals, USA) was applied to the established indirect co-culture model at a concentration of 1 mM for 12 h. Following treatment, protein expression and chemosensitivity were assessed using WB and the CCK-8, respectively, as detailed in Sect. 2.4 and 2.8.

### Measurement of IL-6 levels and functional validation by enzyme-linked immunosorbent assay and WB

The levels of IL-6 in the culture medium were measured using a commercial enzyme-linked immunosorbent assay (ELISA) kit (Human Interleukin 6, IL-6 ELISA KIT, CSB-E04638h, Cusabi, China), according to the manufacturer’s instructions. Samples were collected from the THP-1 cultures, MSCs monocultures, and MSCs-THP-1 co-cultures. The resulting culture medium was then added to wells that had been pre-coated with monoclonal antibodies directed against IL-6. Following three washes with PBS containing 0.05% Tween-20, peroxidase-coupled avidin and a biotinylated anti-IL-6 antibody were added. The level of IL-6 was determined by measuring the absorption at 450 nm using a microplate reader.

To further confirm the functional role of IL-6 in PI3K/Akt signaling, THP-1 cells were treated with recombinant human IL-6 (50 ng/mL, HY-P7044, MedChemExpress, USA) for 24 h. After treatment, cells were harvested, and protein extracts were subjected to WB analysis to evaluate the phosphorylation levels of PI3K and Akt.

### Data processing and statistical analyses

All experimental data were presented as mean ± standard deviation (SD). Further statistical analyses were performed using GraphPad Prism 8.0 (GraphPad Software, USA). For comparisons involving more than two groups, the ANOVA was conducted, followed by post-hoc analysis (e.g., Tukey’s test). Comparisons between two groups were performed using a two-tailed Student’s *t*-test and paired *t*-test. The *p*-value of ≤ 0.05 was considered statistically significant for all tests.

## Results

### Morphological characteristics, cellular functions, and phenotypic profiles of the AML-MSCs in vitro

**Fig. 1:**
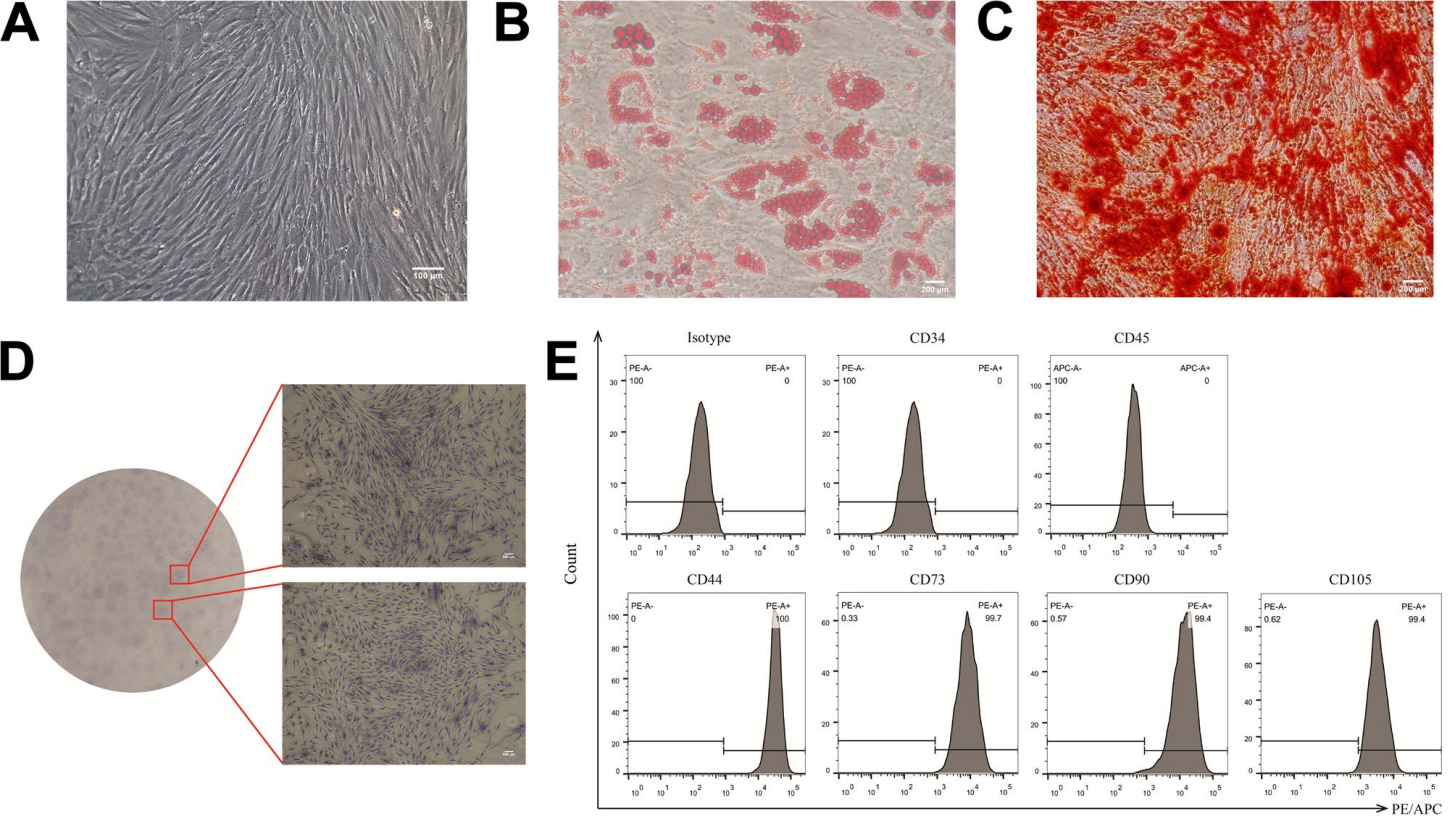
The MSCs identification. (**A)** Morphological characteristics of the MSCs under the microscope (scale bar = 100 μm). **(B)** Oil Red O stained the adipogenically differentiated MSCs (scale bar = 200 μm). **(C)** Alizarin Red S stained the osteogenically differentiated MSCs (scale bar = 200 μm). **(D)** CFU-C assay of the MSCs (scale bar = 500 μm). **(E)** Flow cytometry profiles of surface marker expression on the MSCs. The isotype panel used isotype-matched control antibodies as a reference for background fluorescence, ensuring accurate interpretation of specific antigen staining rather than representing unstained samples

The MSCs were isolated from AML bone marrow and then cultured. Microscopic analysis revealed that the cultures exhibited the characteristic shuttle morphology of the MSCs, with a swirling growth pattern during the primary culture phase (Fig. 1A). To assess their differentiation potential, the MSCs were induced to differentiate into osteogenic and adipogenic lineages. The stability of these differentiated states was confirmed by Oil Red O staining for lipid droplets and Alizarin Red S staining for calcified nodules (Fig. 1B, C). The MSCs also displayed favorable proliferation capabilities, as demonstrated by the colony-forming assay (Fig. 1D). To verify the phenotype of the isolated MSCs, flow cytometry analysis was performed. The analysis confirmed the positive expression of mesenchymal markers CD44, CD73, CD90, and CD105, and the negative expression of hematopoietic markers CD34 and CD45 (Fig. 1E). These MSCs that maintained normal cellular functions and correct phenotypes following adipogenic induction were subsequently used in co-culture experiments with AML cells.

### Co-culture reduces the chemosensitivity of AML cells to DNR and Ara-C

**Fig. 2:**
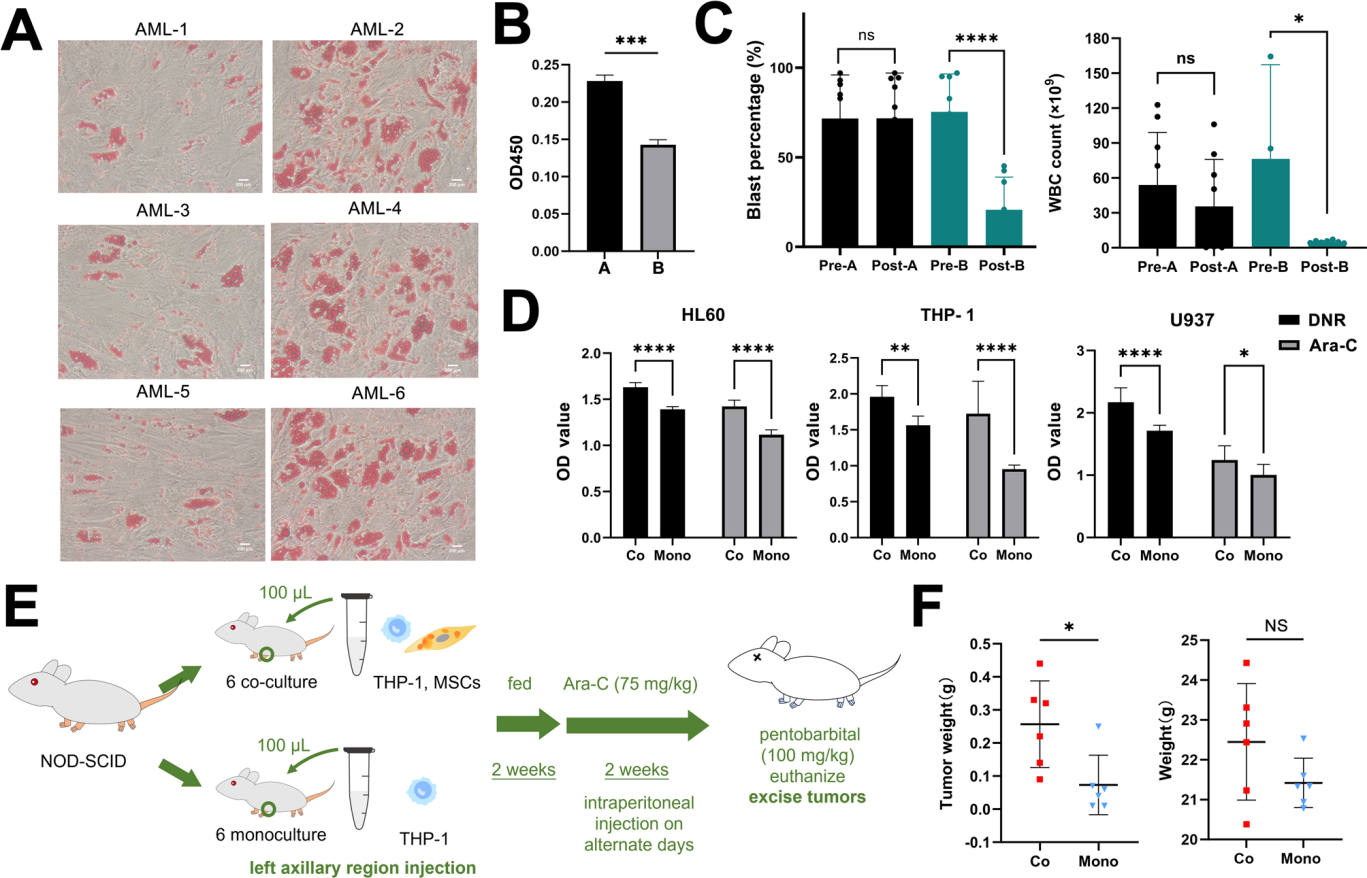
The MSCs with adipogenic differentiation potential reduce sensitivity of AML cells to DNR and Ara-C. (**A**) Oil Red O stained adipogenical differentiation potential MSCs from AML patients (scale bar = 200 μm). (**B**) Isopropanol dissolves lipid droplet in the adipogenically differentiated MSCs and the absorbance was measured at 450 nm (A group: AML2, 4, and 6; B group: AML1, 3, and 5). (**C**) Comparison of bone marrow blast percentage and peripheral WBC counts before and after initial treatment in groups A and B. Statistical significance determined by a paired Student’s *t*-test. Pre-, before initial treatment; Post-, after initial treatment. (**D**) After co-culture with the adipogenically differentiated MSCs, the chemosensitivity of AML cells to DNR and Ara-C was detected by CCK-8. Using the dual-wavelength method, the absorbance was measured at 450 nm and 630 nm. OD Value, the value of OD450-OD630. (**E**) The xenograft experiment flow chart. (**F**) Quantitative comparison of tumor weight and mice weight between co-culture and monoculture group. Statistical significance is denoted as *p < 0.05, **p < 0.01, ***p < 0.001, ****p < 0.0001, determined by an unpaired Student’s *t*-test. Co, co-culture group; Mono, monoculture group

To evaluate the malignant phenotypes of the MSCs, we compared the MSCs from AML patients and healthy donors. Morphology, surface marker expression, proliferative capacity, and differentiation potential were assessed. No substantial differences were observed between the two groups, except for adipogenic differentiation capacity (Figure S1 in the Supplementary Materials). The MSCs from AML patients showed varying degrees of adipogenic differentiation (Fig. 2A). Some MSCs populations exhibited a clear propensity for adipogenesis (Fig. 2B). Clinical follow-up revealed that the chemotherapy efficacy and prognoses of AML patients associated with these MSCs were not favorable. There were no significant differences in age between patients with high and low adipogenic differentiation of MSCs (Figure S2). Paired *t*-tests were performed to evaluate the proportions of bone marrow blast cells and peripheral white blood cell (WBC) counts before and after chemotherapy in both patient groups. Patients with higher adipogenic differentiation exhibited no significant reduction in the proportion of bone marrow blast cells following chemotherapy (Fig. 2C). Conversely, patients with lower adipogenic differentiation demonstrated a significant reduction in bone marrow blast cells post-treatment. A similar trend was observed in peripheral WBC counts (Fig. 2C). These analyses indicated that patients with high adipogenic differentiation of MSCs exhibited a poor response to chemotherapy.

Therefore, these MSCs were selected for indirect co-culture with AML cells, including THP-1, U937, and HL-60 cell lines. After treatment with DNR and Ara-C, cell viability was compared to that in monoculture to assess the chemosensitivity of the AML cell lines. The results showed that AML cells co-cultured with the adipogenically differentiated MSCs had reduced chemosensitivity (Fig. 2D). Subsequently, an engrafted NOD-SCID mice model was established (Fig. 2E). In this model, the co-culture group received simultaneous injections of the MSCs and THP-1 cells, while the control group received THP-1 cells alone. Two weeks after cell injection, tumors were established. The mice subsequently received Ara-C treatment for two weeks, after which tumors were harvested. The results indicated that the co-culture group exhibited larger tumor size and weight compared to the control group, while no significant change in the self-weight was observed (Fig. 2F, and Figure S3). These results in vitro and in vivo demonstrated that AML cells co-culturing with the adipogenically differentiated MSCs reduce their chemosensitivity.

### Altered metabolic pathways related to the drug sensitive of co-cultured AML

**Fig. 3:**
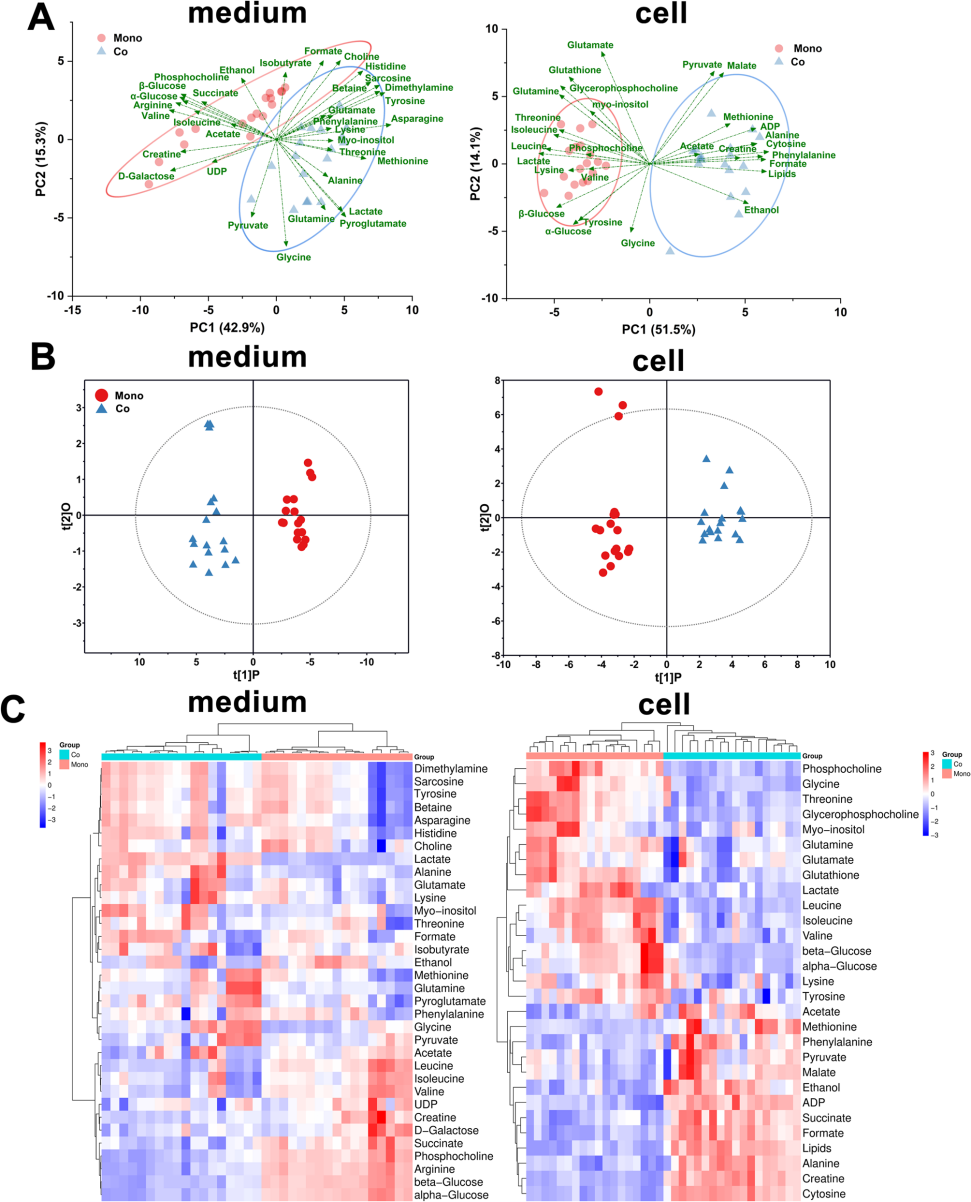
Metabolomics pattern recognition analysis and cluster analysis. (**A)** PCA biplot of ^1^H NMR assignment data for metabolites in medium and cells. **(B)** The score plots of OPLS-DA depict the discrimination between co-culture and monoculture group in medium (R^2^X = 0.858, R^2^Y = 0.949, Q^2^ = 0.921, *p* < 0.0001), and in cells (R^2^X = 0.731, R^2^Y = 0.9953, Q^2^ = 0.938, *p* < 0.0001). **(C)** Heatmap depicting clustering analysis results for metabolites in cell extracts and culture medium. Co, co-culture group; Mono, monoculture group

To further explore the underlying mechanisms of the metabolism, the NMR-based metabolomics analysis of AML cells was performed to clarify the metabolic profiles affected by the adipogenically differentiated MSCs. Given the comparable trends observed among THP-1, U937, and HL-60 cells in chemosensitivity experiments, THP-1 was selected for follow-up studies due to its stable growth characteristics and well-established utilization. Once the NMR spectra were subjected to the standard preprocessing, the entire ^1^H NMR spectral datasets were directly put into the statistical software for unsupervised PCA. The 3D PCA score plots, derived from the full spectrum, displayed clear separations between the groups in both medium-based and cellular metabolic profiles (Figure S4). Kinds of low-weight metabolites from cells and culture medium were identified according to the HMDB database. The results demonstrated 29 and 33 metabolites in the cells and culture medium, respectively (Figure S5). Detailed assignments were documented in Table S1 and S2 in the Supplementary Materials, and the relative contents were calculated based on the area under the peaks of the normalized spectra. Based on the relative contents, the biplots of PCA were performed to identifying specific metabolites that drive the group differences (Fig. 3A). In the medium-based biplot, metabolites such as alanine, glycine, and lactate aligned with the co-culture group, indicating their increased relative abundance in the extracellular environment due to interactions with the MSCs. Conversely, metabolites like acetate and glucose were more closely associated with the monoculture group. In the cellular biplot, intracellular metabolites such as alanine and phenylalanine were associated with the co-culture group, whereas glutamate, glutamine, and phosphocholine were linked to the monoculture group. These biplots not only confirmed the PCA score plots but also highlighted the key metabolites driving the observed metabolic differences.

**Fig. 4:**
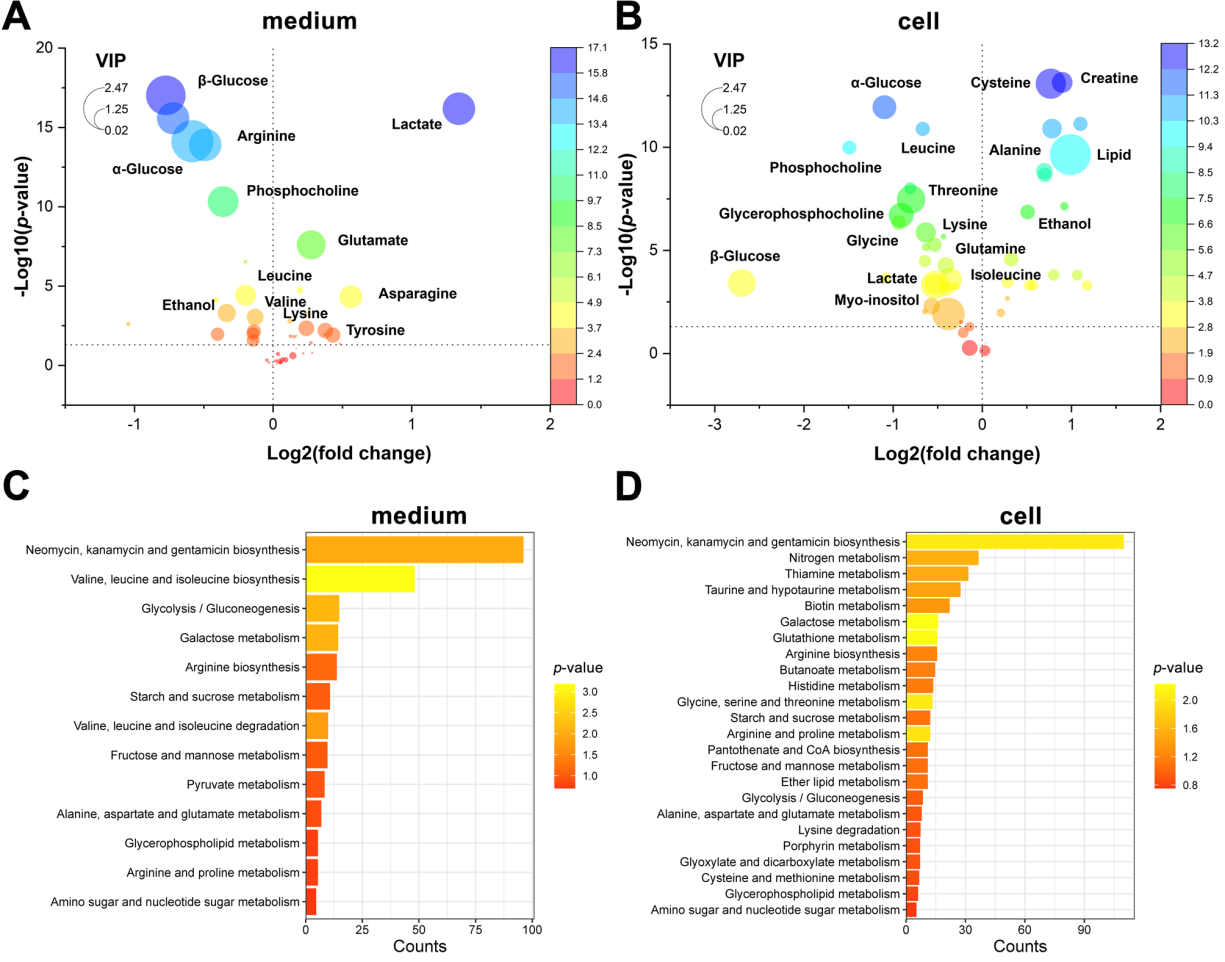
Metabolomics pattern recognition analysis and cluster analysis. (**A**,** B)** The volcano plots of the metabolites in medium and cells. The color and size of the labels in the volcano plots denote the magnitude of *p*-value and VIP values, respectively. Warmer colors and larger label sizes indicate higher values. The dotted line on the Y-axis delineates cases with *p* = 0.05. **(C**,** D)** Co-culture and monoculture groups differential metabolites KEGG enrichment pathway diagram. Statistical significance is determined by an unpaired Student’s *t*-test

After initially identified the statistically significant metabolites, the supervised OPLS-DA were performed for further screening of differential metabolites. The score plot of OPLS-DA displayed a robust model verified by cross validation results, confirming the validity and not excessively fitting (Fig. 3B, and Figure S6). The heatmap of relative contents of identified metabolites in the cells and culture medium also displayed similar results with PCA and OPLS-DA (Fig. 3C), providing clear representation of the metabolic profiles and differential metabolites in each sample. Subsequently, the VIP was set > 1, along with *p*-value < 0.05, were used to screen differential metabolites. The volcano plots were generated by integrating the fold change values, *p*-values from univariate analysis, and VIP values from multivariate analysis (Fig. 4A, B). The results intuitively showed that the levels of several metabolites including alanine, creatine, cysteine, and lipid in co-culture THP-1 were significantly elevated compared to those in monoculture THP-1 (*p* < 0.05). The levels of lactate, leucine, isoleucine, threonine and glycine were higher in monoculture THP-1 (*p* < 0.05). Besides, in the culture medium, co-culture THP-1 exhibited increased levels of lactate, glutamate, asparagine, and tyrosine (*p* < 0.05), while the levels of glucose, arginine, leucine, and phosphocholine were decreased (*p* < 0.05). All the identified differential metabolites (*p* < 0.05 and VIP > 1) were then used in a pathway enrichment analysis by Metaboanalyst 6.0. The result revealed the altered pathways related to glycolysis, glutamine and glutamate metabolism, alanine, aspartate, and glutamate metabolism, glycine, serine and threonine metabolism, glutamine metabolism, and glycogen synthesis (Fig. 4C, D).

### The MSCs enhance the chemosensitivity of AML cells by regulating environmental information processing pathway

**Fig. 5:**
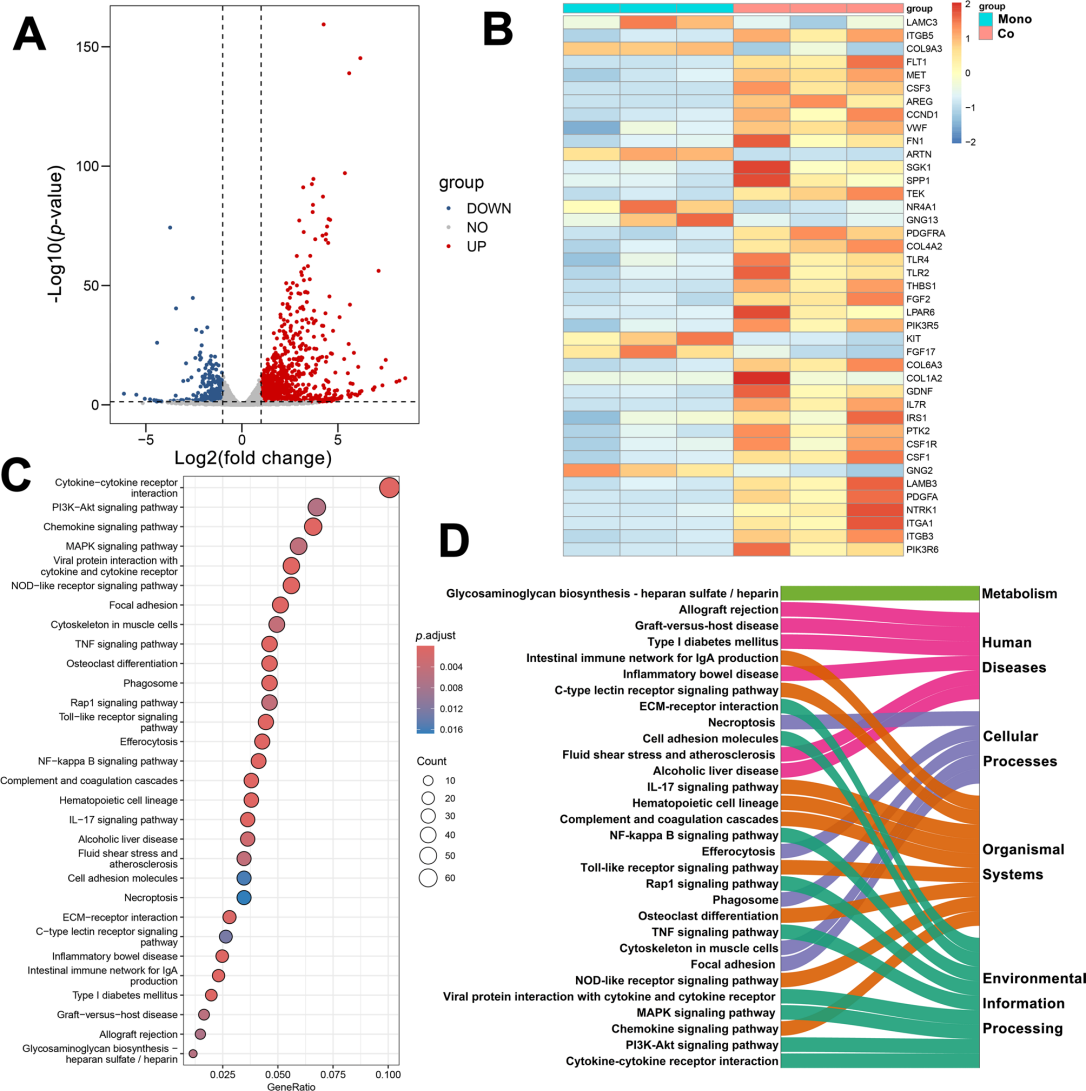
Transcriptome analysis of DNR and Ara-C sensitivity-related co-culture. (**A)** Volcano plots of the co-culture versus the monoculture group (*p* < 0.0001, |log2(fold change) |>5). **(B)** Heatmap of the relative content of the top 40 DEGs between the co-culture and monoculture groups. Co, co-culture group; Mono, monoculture group. **(C)** Co-culture and monoculture groups differential gene KEGG enrichment pathway diagram (*p* < 0.05, Count > 10). **(D)** KEGG enrichment pathway classification of DEGs

Metabolomic analysis revealed alterations in metabolic phenotypes, which were found to be directly associated with the low chemosensitivity phenotype. However, because metabolism represents the most downstream outcome of various regulatory pathways, metabolic changes can be influenced by multiple factors. Transcriptomics can alternatively help identify the upstream genetic or regulatory drivers responsible for the metabolic alterations. Total mRNA was extracted from THP-1 cells, and transcriptomic data were obtained using NGS. A heatmap was generated to visualize the correlation patterns of transcriptomic profiles between THP-1 cells monocultured and those co-cultured with the MSCs (Figure S7). Within-group correlations were notably high, while between-group correlations were slightly lower. Such results indicated a high level of reproducibility among samples and displayed the transcriptomic alterations in THP-1 cells induced by the adipogenically differentiated MSCs co-culture. To further identify DEGs that regulate the metabolic alterations observed in co-cultured THP-1, the volcano plot was constructed (Fig. 5A). According to the *p*-value and absolute value of the Log2(fold change) to screen DEGs, the threshold was set at 0.05 and 1 respectively. 1323 genes were screened, consisting of 343 significantly down-regulated DEGs and 980 significantly up-regulated DEGs. For clarity and effective presentation, the top 40 DEGs (*p* < 0.0001, |log2(fold change)|>5) were selected for display in the heatmap (Fig. 5B). In THP-1 cultured with the MSCs, top ranking DEGs tended to be upregulated in contrast to monoculture, including *ITGB5*,* LAMB3*,* CSF1*,* PDGFA*, and *PIK3R6*. Furthermore, *LAMC3*,* ARTN*,* KIT*, and a small number of other genes have been observed to demonstrate a downward trend.

Further KEGG pathway enrichment analysis was performed on DEGs between the co-culture group and monoculture group. As illustrated in Fig. 5C, the top 30 (*p* < 0.05, count >10) in enrichment significance were displayed. Although pathways in complex cellular processes cannot be strictly divided into single categories, the KEGG functional classification was applied to the enrichment results [28, 29]. This standardized, hierarchical system groups pathways into seven major categories—Metabolism, Genetic Information Processing, Environmental Information Processing, Cellular Processes, Organismal Systems, Human Diseases, and Drug Development—providing clearer interpretation of high-throughput data. The top 30 pathways in our data were fell into five of these categories (Fig. 5D). Among them, MSCs co-culture primarily affected pathways classified under Environmental Information Processing in KEGG, including the PI3K-Akt signaling pathway, the TNF signaling pathway, the NF-κB signaling pathway, and cytokine-cytokine receptor interaction. These pathways function at the interface between the cell and its external environment, receiving, transmitting and integrating extracellular signals. Notably, AML cells were cultured in an indirect co-culture system with MSCs, indicating that the observed transcriptional changes were driven by MSCs-induced microenvironmental modulation. This subsequently led to the associated alterations in metabolism and chemosensitivity to DNR and Ara-C. Other enriched pathways, including those classified under Organismal Systems and Cellular Processes, were interconnected with the Environmental Information Processing pathways altered by MSCs, collectively contributing to the reduced chemosensitivity observed in THP-1 cells.

### Inhibition of PI3K/Akt signaling pathway increases the AML chemosensitivity to DNR and Ara-C

**Fig. 6:**
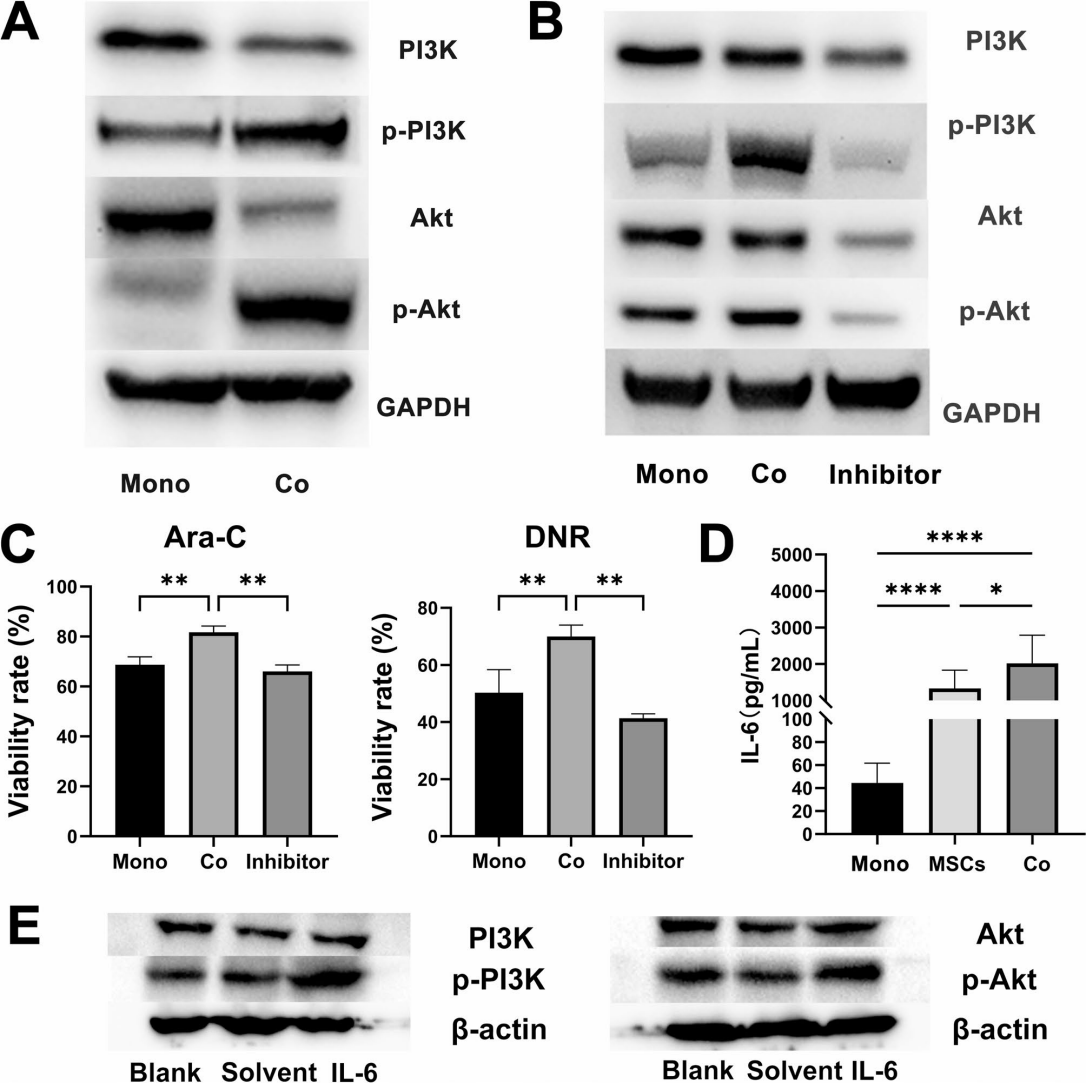
Verification of PI3K/Akt signaling pathway and the inhibitor MK-2206 efficacy. (**A)** Key mediator’s expression of PI3K/Akt signaling pathway in two groups (monoculture and co-culture). **(B)** Key mediator’s expression of PI3K/Akt signaling pathway in three groups (monoculture, co-culture, and addition inhibitor in co-culture). Full-length blots are presented in Supplementary Materials Figure S9. **(C)** The chemosensitivity of THP-1 to DNR and Ara-C was detected by the CCK-8 method. **(D)** IL-6 concentration in medium supernatants was tested in each group by ELISA. **(E)** Key mediator’s expression of PI3K/Akt signaling pathway in THP-1 cells after IL-6 treatment. Full-length blots are presented in Supplementary Materials Figure S10. Statistical significance is denoted as *p < 0.05, **p < 0.01, ****p < 0.0001, determined by ANOVA. Co, co-culture group; Mono, monoculture group

**Fig. 7:**
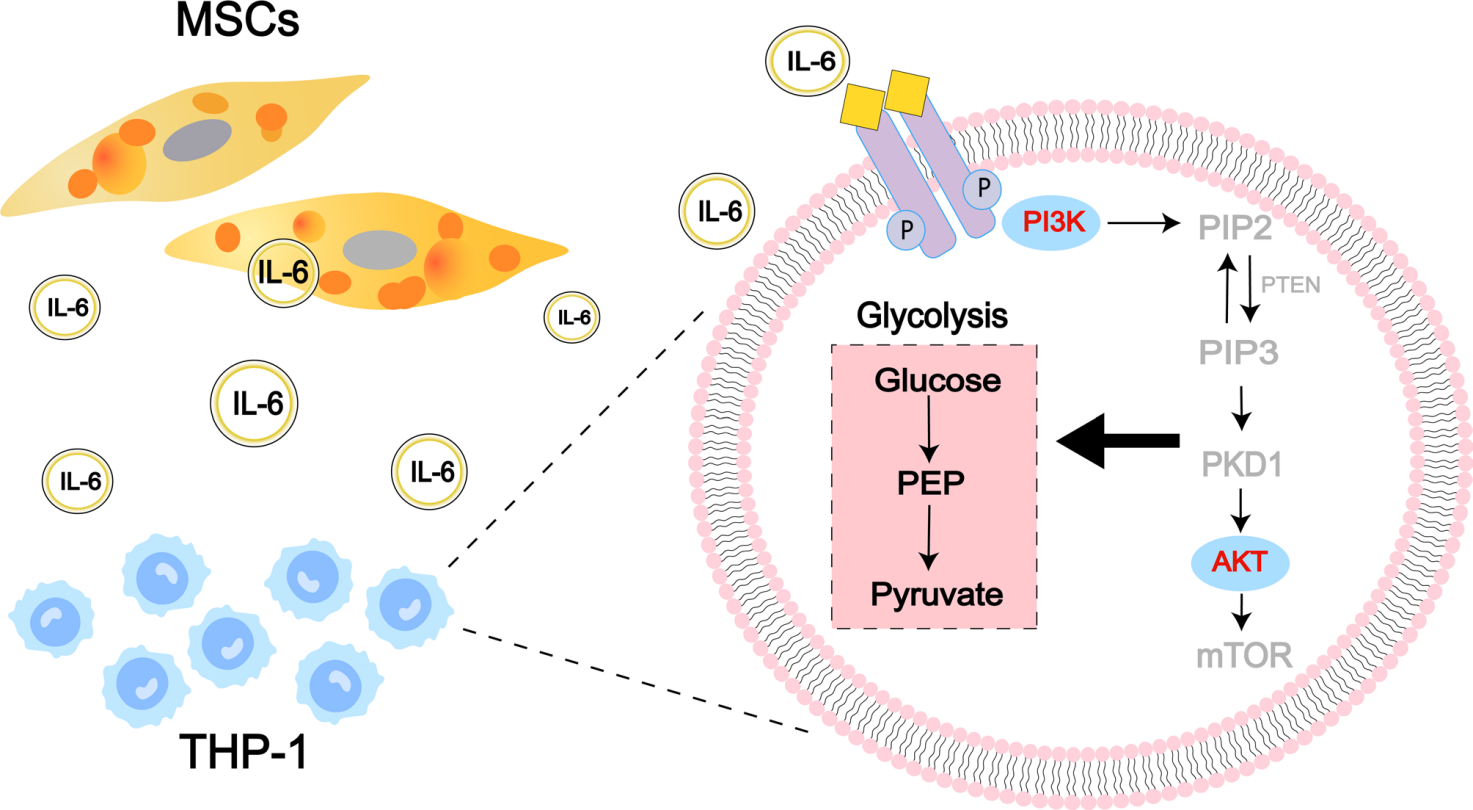
The mechanism diagram of affected THP-1 by the adipogenically differentiated MSCs

Metabolomics analysis revealed that glycolysis was significantly altered in THP-1 cells following co-culture with the MSCs. Transcriptomic analysis subsequently indicated notable changes in the PI3K/Akt signaling pathway in THP-1 cells after co-culture. Given the central role of PI3K/Akt signaling in glycolysis, the MSCs may protect AML cells from chemotherapy through modulation of this pathway. To verify PI3K/Akt activation after co-culture, key mediators were examined using WB. Increased phosphorylation of PI3K and Akt (p-PI3K and p-Akt) in co-cultured THP-1 cells was observed (Fig. 6A). To further evaluate whether enhanced drug resistance was mediated by this pathway, the selective Akt phosphorylation inhibitor MK-2206 was used. CCK-8 assays showed that MK-2206 alone, at 0.5 and 1.0 µM, did not significantly affect AML cell viability, whereas 2.0 µM produced modest inhibition (Figure S8). Therefore, 1.0 µM was chosen for subsequent studies. Treatment with MK-2206 significantly reduced p-PI3K and p-Akt levels in THP-1 cells (Fig. 6B). Notably, lower cell viability rate indicated the MK-2206 treatment markedly increased THP-1 cell sensitivity to Ara-C and DNR (Fig. 6C). Also, the IL-6 levels were measured by ELISA according to previous reports about its role in MSCs-derived IL-6 mediates tumor progression [30–32], and as the key regulator of PI3K/Akt signaling [16, 33, 34]. The IL-6 concentrations were significantly higher in co-culture medium compared with THP-1 or MSCs monocultures (Fig. 6D). To further validate the functional role of IL-6, we treated THP-1 cells with recombinant human IL-6 and examined PI3K pathway activation. WB revealed that IL-6 treatment markedly enhanced PI3K and Akt phosphorylation compared with both the blank and solvent control groups (Fig. 6E), thereby confirming its contribution to PI3K signaling activation under co-culture conditions. Finally, as shown in Fig. 7, an integrated schematic summarizes alterations observed in co-cultured THP-1 cells based on metabolomics, transcriptomics, and protein expression analyses.

## Discussion

The MSCs play a pivotal role in the progression of AML, with the ability to regulate and interact with AML cells through various mechanisms, including the secretion of factors, cytokines, metabolites, and exosome [35, 36]. These behaviors alter the BMM, enhancing the protection and support of AML cells. A co-culture model was established by utilizing the MSCs with higher adipogenically differentiated potential from AML patients, so as to investigate how these induced adipogenically differentiated MSCs enhance the survival and protection of AML cells. In the cell model, co-culture group AML cells had lower chemotherapy sensitivity. And in the animal model, those mice vaccinated with both MSCs and AML cells exhibited larger tumor volume and mass following treatment with Ara-C and DNR. These findings indicated that adipogenic differentiation of the MSCs prevented the action of chemotherapy on AML cells. Consequently, a preliminary exploration of the underlying protective mechanism was conducted through the integration of metabolomics and transcriptomics analyses.

The study of the influence from the tumor microenvironment to cancer cells in terms of metabolism has become a new and exciting field of cancer research. Tumor metabolism is characterized by high levels of glucose dependence and enhanced glycolytic metabolic activity [37, 38]. In this work, metabolomics analysis revealed that glycolysis is one of the key metabolic pathways that are significantly altered in AML cells during co-culture with the adipogenically differentiated MSCs. Venetoclax (VEN) and azacitidine (AZA)-resistant AML cell lines established in a previous study exhibited increased lactate levels and glycolytic activity compared with non-resistant cells, and glycolysis inhibition using 2-deoxy-D-glucose restored their sensitivity to VEN + AZA treatment [39]. It was assumed that such low sensitivity was caused by the adipogenically differentiated MSCs. However, they displayed the same glycolysis characteristic as other mechanism. This may indicate that targeting glycolysis could be a universal way to overcome chemotherapy resistance in AML therapy.

The results of the transcriptomics indicated that the role of the adipogenically differentiated MSCs in regulating glycolysis in AML cells appeared to be mediated through specific signaling pathways. The Environmental Information Processing pathway was significantly changed in AML cells after co-culture, particularly the PI3K/Akt signaling pathway, which presented a high enrichment level. When considered in conjunction with the metabolomics results, this may suggest that the adipogenically differentiated MSCs alter glycolysis in AML cells via the PI3K-Akt signaling pathway, thereby hindering the effectiveness of Ara-C and DNR therapy. As a classic pathway, the PI3K/Akt signaling pathway was widely involved in various disease [40, 41]. In the context of AML, this pathway is one of the aberrantly upregulated intracellular pathways and plays a critical role in leukemogenesis while being central to metabolic regulation [42–44]. Indeed, the energy metabolism of AML cells may potentially rely on its downstream signaling [45, 46]. A transcription factor, GLI1, led to an increase in the growth of AML cells and a decrease in Ara-C and adriamycin (ADR) sensitivity [47]. This effect was mediated by the activation of the PI3K/Akt signaling pathway, promoting glycolysis and cell-cycle progression through the regulation of key downstream targets, such as cyclins and metabolic enzymes. Others have reported that targeting the PI3K/Akt/mTOR pathway could overcome VEN chemoresistance in AML [48]. Therefore, the PI3K/Akt signaling pathway is inferred to be involved in MSCs-mediated reductions in chemosensitivity of AML cells. Further quantitative analyses of key proteins in the PI3K/Akt signaling pathway and the chemosensitivity assays of AML cells validated this assumption.

Besides, the transcriptomic results also indicated that other glycolysis-associated signaling pathways were involved in the MSCs-mediated chemoresistance in AML. Among these pathways, the NF-κB signaling pathway functions as a mediator of Environmental Information Processing in cells. Nuclear factor erythroid 2-related factor 2 (Nrf2) is a transcription factor associated with NF-κB signaling pathway. It enhances glycolysis by promoting the expression of genes involved in glucose uptake and metabolism [49, 50]. Recent studies reported that pharmacological inhibition of Nrf2 by brusatol significantly reduces glycolytic activity in AML cells. It decreased glucose consumption and lactate production, sensitizing AML cells to Ara-C both in vitro and in vivo [51]. Additionally, elevated glycolysis due to Nrf2 overexpression was linked to VEN resistance in AML cells [52]. These researches suggested that the MSCs may affect the NF-κB signaling pathway of AML cells through Nrf2, and targeting the Nrf2-mediated glycolytic pathway could provide a therapeutic strategy to overcome MSCs-induced chemoresistance. Although NF-κB signaling was not directly interrogated in this study, IL-6, a cytokine that was involved in both NF-κB and the confirmed PI3K/Akt pathways, was elevated in the co-culture medium, providing indirect support for potential NF-κB involvement. As a key cytokine in the cytokine-cytokine receptor interaction pathway, the IL-6 activates the PI3K-Akt signaling pathway and forms a positive feedback loop with the NF-κB signaling pathway [53–55]. All of these pathways were enriched in the KEGG analysis of the current work, suggesting increased IL-6 levels as a critical role in the MSCs regulation of AML cell metabolism and chemosensitivity.

Eventually, the MSCs have been observed to interact with AML cells, thereby influencing a range of biological processes that are implicated in tumor progression and chemotherapy resistance. Changes in metabolism and signaling pathways provide insights into the MSCs-mediated regulation of AML cells. These findings suggest potential therapeutic targets for overcoming chemotherapy resistance. However, several limitations of this study should be noted. The absence of a healthy donor MSCs control group limits the ability to distinguish general MSCs effects from those driven by adipogenesis, and future inclusion of healthy donor MSCs will be important to address this gap. In addition, MSCs may not only mediate chemoresistance but also promote AML engraftment or growth without drug pressure, indicating the need for proper controls to separate these roles. Finally, although a subcutaneous xenograft model allowed reproducible experiments, it does not fully simulate the bone marrow niche, and further studies will focus on using improved humanized or marrow-engrafted models to better capture disease biology.

By recognizing these limitations and addressing them in subsequent studies, a more comprehensive understanding of how MSCs contribute to AML progression and therapy resistance can be expected. This, in turn, may help identify more effective strategies for targeting the bone marrow niche in AML treatment. In the future, the relationship between transcriptomic and metabolomic alterations should be clarified, and the role of IL-6 in MSC–AML interactions during chemotherapy should be further investigated. Future research should focus on delineating MSCs-mediated metabolic and transcriptional changes in AML cells. In addition, IL-6-related signaling pathways, particularly the PI3K/Akt pathway, should be further explored to better understand their role in reducing chemosensitivity. Gene knockdown or pharmacological inhibition of IL-6 signaling could help validate these pathways as potential therapeutic targets.

## Conclusions

In summary, this work demonstrates that adipogenically differentiated MSCs enhance AML cell survival and chemoresistance by modulating metabolic and signaling pathways. The PI3K/Akt signaling pathway and IL-6 were suggested to play key roles in this process. These findings provide new insights into MSC–AML interactions and highlight the regulation of AML by MSCs as a potential therapeutic target. Further research should focus on elucidating the underlying molecular mechanisms and on evaluating potential strategies to disrupt MSCs-mediated chemoresistance. Such efforts may facilitate the development of novel therapeutic approaches for AML.

## Supplementary Information

Below is the link to the electronic supplementary material.

Supplementary Material 1

## Data Availability

Transcriptomics (RNA-seq) raw data have been deposited in the National Center for Biotechnology Information Sequence Read Archive (NCBI SRA) database ([https://www.ncbi.nlm.nih.gov/bioproject/](https:/www.ncbi.nlm.nih.gov/bioproject)), under accession number PRJNA1259224: [https://dataview.ncbi.nlm.nih.gov/object/PRJNA1259224](https:/dataview.ncbi.nlm.nih.gov/object/PRJNA1259224). The data generated or analyzed during this study are included in this article and its Supplementary Materials, or if not available, are available from the corresponding author upon reasonable request.

## Declarations

### Declaration of AI and AI-assisted technologies in the writing process

The authors declare that they have not used AI-generated work in this manuscript.

## Abbreviations

AML: Acute myeloid leukemia
Ara-C: Cytarabine
BMM: Bone marrow microenvironment
CCK-8: Cell counting kit-8
cDNA: Complementary DNA
DEGs: Differentially expressed genes
DNR: Daunorubicin
ELISA: Enzyme-linked immunosorbent assay
FBS: Fetal bovine serum
IL-6: Interleukin-6
KEGG: Kyoto Encyclopedia of Genes and Genomes
MSCs: Mesenchymal stem cells
NGS: Next-generation sequencing
OPLS-DA: Orthogonal partial least squares with discriminant analysis
PCA: Principal components analysis
PE: P-phycoerythrin
P-gp: P-glycoprotein
PS: Penicillin-streptomycin
SD: Standard deviation
TSP: 2,2,3,3-d-(4)-3-(trimethylsilyl) propionate
VIP: Variable importance projection
WB: Western blotting
WBC: White blood cell
