# Supplementary material for: Integrated metabolomic and transcriptomic analysis identifies adipogenic differentiation of mesenchymal stem cells as a driver of chemoresistance in acute myeloid leukemia
Source: J Exp Clin Cancer Res. 2025 Oct 17;44:291. doi: 10.1186/s13046-025-03550-0 (PMC12534991; doi:10.1186/s13046-025-03550-0)
Supplement: Supplementary file 1 — Supplementary Material 1 [file 13046_2025_3550_MOESM1_ESM.docx]

**Supplementary Materials**

**Integrated metabolomic and transcriptomic analysis identifies adipogenic differentiation of mesenchymal stem cells as a driver of chemoresistance in acute myeloid leukemia**

Zhipeng Pan ^1, 2, #^, Rong Hu ^1, 2, #^, Dandan Li ^1^，Siwen Deng ^1, 2^, Haishan Yi ^1, 2^, Zhengwei Duan ^3^, Lixia Kang ^4^, Ling Chen ^5^, Mengyao Wang ^1^, Yue Duan ^1^, Xiaofan Jia ^1^, Pengfei Guo ^6^, Yang Chen ^1, 2, *^

**Author affiliation**

^1^ Department of Laboratory Medicine, Fujian Medical University, Fuzhou 350122, China

^2^ Key Laboratory of Clinical Laboratory Technology for Precision Medicine (Fujian Medical University), Fujian Province University, Fujian Medical University, Fuzhou 350122, China

^3^ Department of Clinical Laboratory, Sir Run Run Shaw Hospital, Zhejiang University School of Medicine, Hangzhou 310016, China.

^4^ Department of Clinical Laboratory, Fujian Medical University Union Hospital, Fuzhou 350001, China

^5^ Fuzhou Second General Hospital, Fuzhou 350007, China

^6^ Department of Electronic Science, Fujian Provincial Key Laboratory of Plasma and Magnetic Resonance, Xiamen University, Xiamen 361005, China

^*^ **Correspondence**

Yang Chen, Department of Laboratory Medicine, Fujian Medical University, Fuzhou 350122, China. Email: chzhy85@fjmu.edu.cn.

^#^ Zhipeng Pan and Rong Hu contributed equally to this work.

**
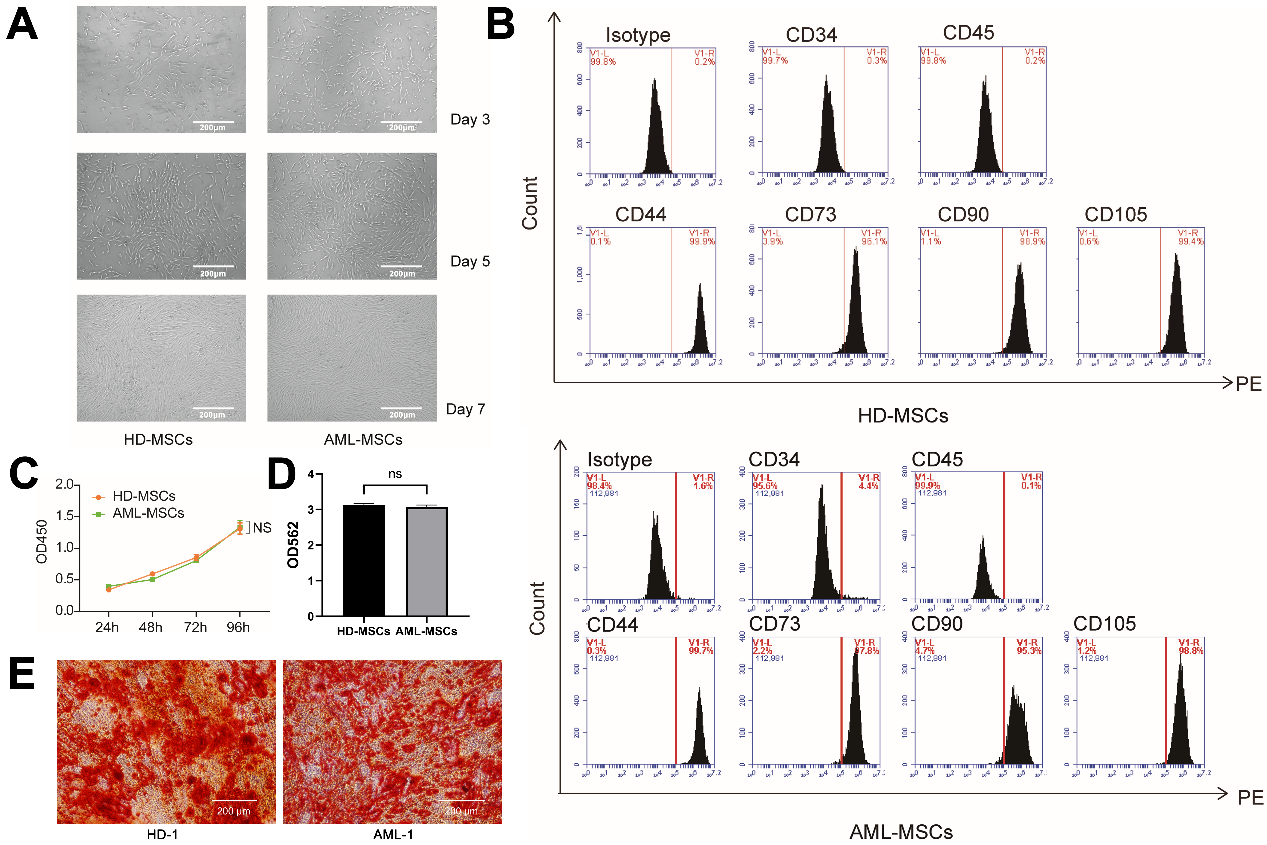
**

**Figure S1 Comparison of MSCs from healthy donors and AML-MSCs. (A)** Morphological characteristics (scale bar=200 μm). **(B)** Flow cytometry profiles of surface marker expression. **(C)** Cell proliferative activity detected by CCK-8. **(D)** After staining with Alizarin Red S, 10% cetylpyridinium chloride dissolved calcified nodules and the absorbance was measured at 562 nm. **(E)** Imaging of Alizarin Red S stained osteogenic differentiated MSCs (scale bar=200 μm). NS, not significant.

**
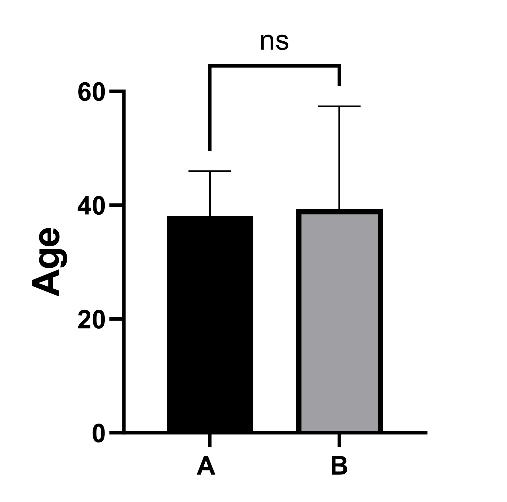
**

**Figure S2 Comparison of patient age between A and B groups.** A group: AML2, 4, and 6; B group: AML1, 3, and 5.

**
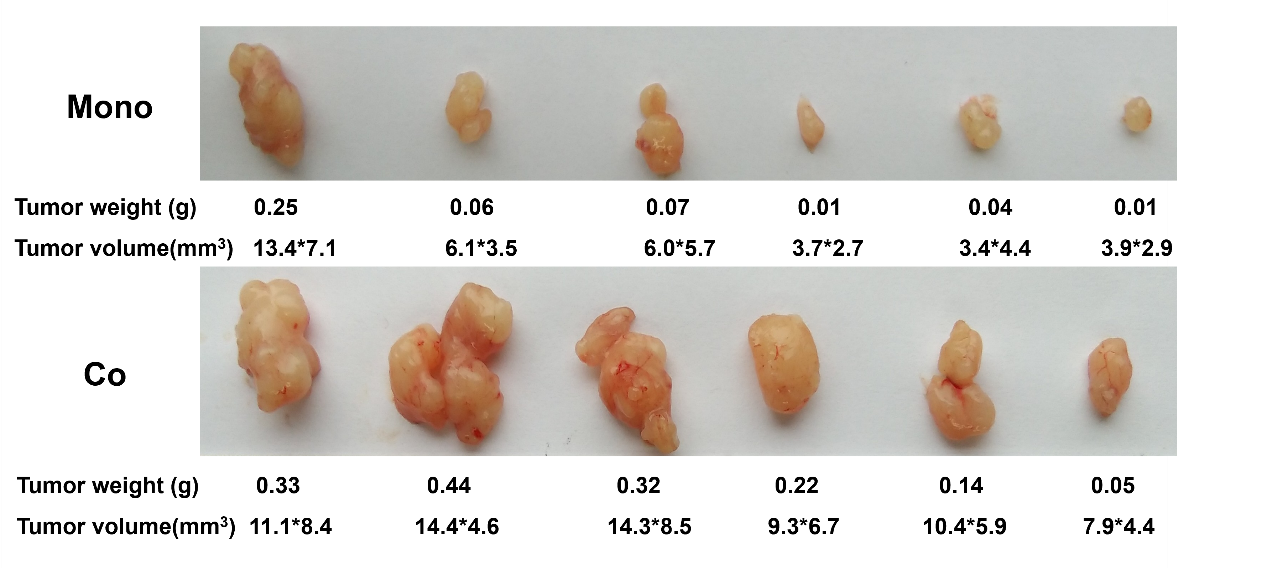
**

**Figure S3** **The tumor weight and volume of the engrafted AML NOD-SCID mice.** Co, co-culture group; Mono, monoculture group.

**
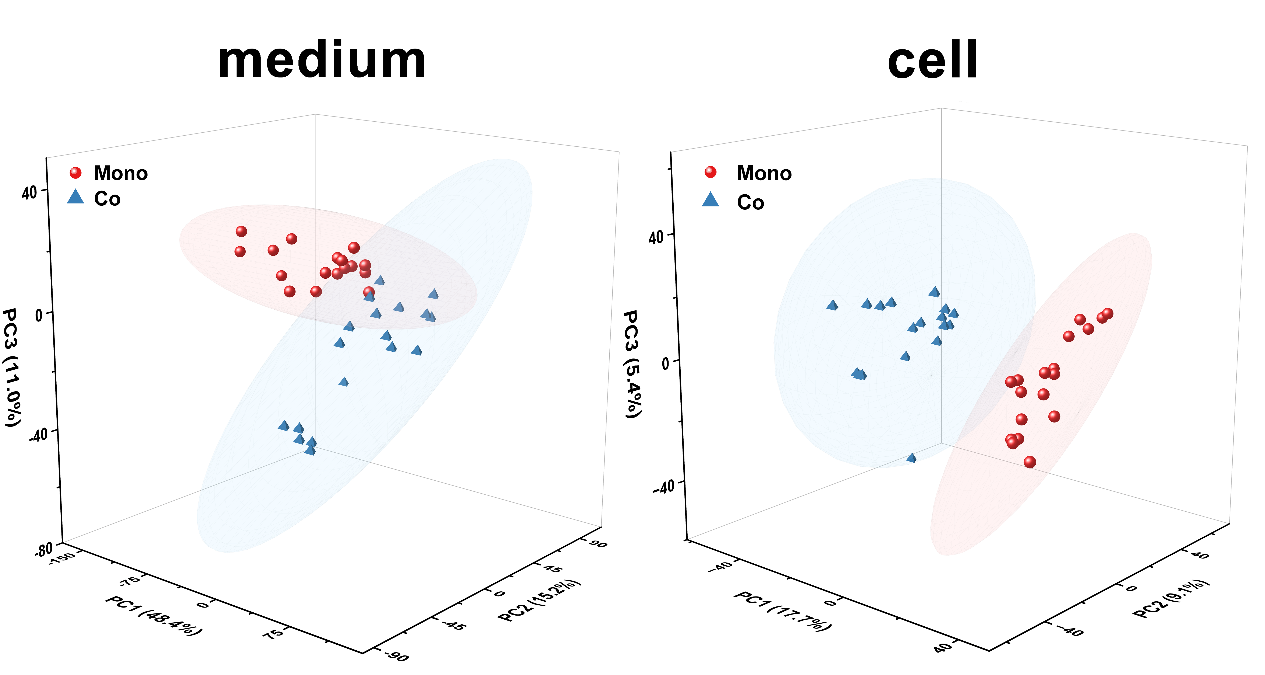
**

**Figure S4** **The 3D score plots of PCA depict the discrimination between co-culture and monoculture group.** Co, co-culture group; Mono, monoculture group.


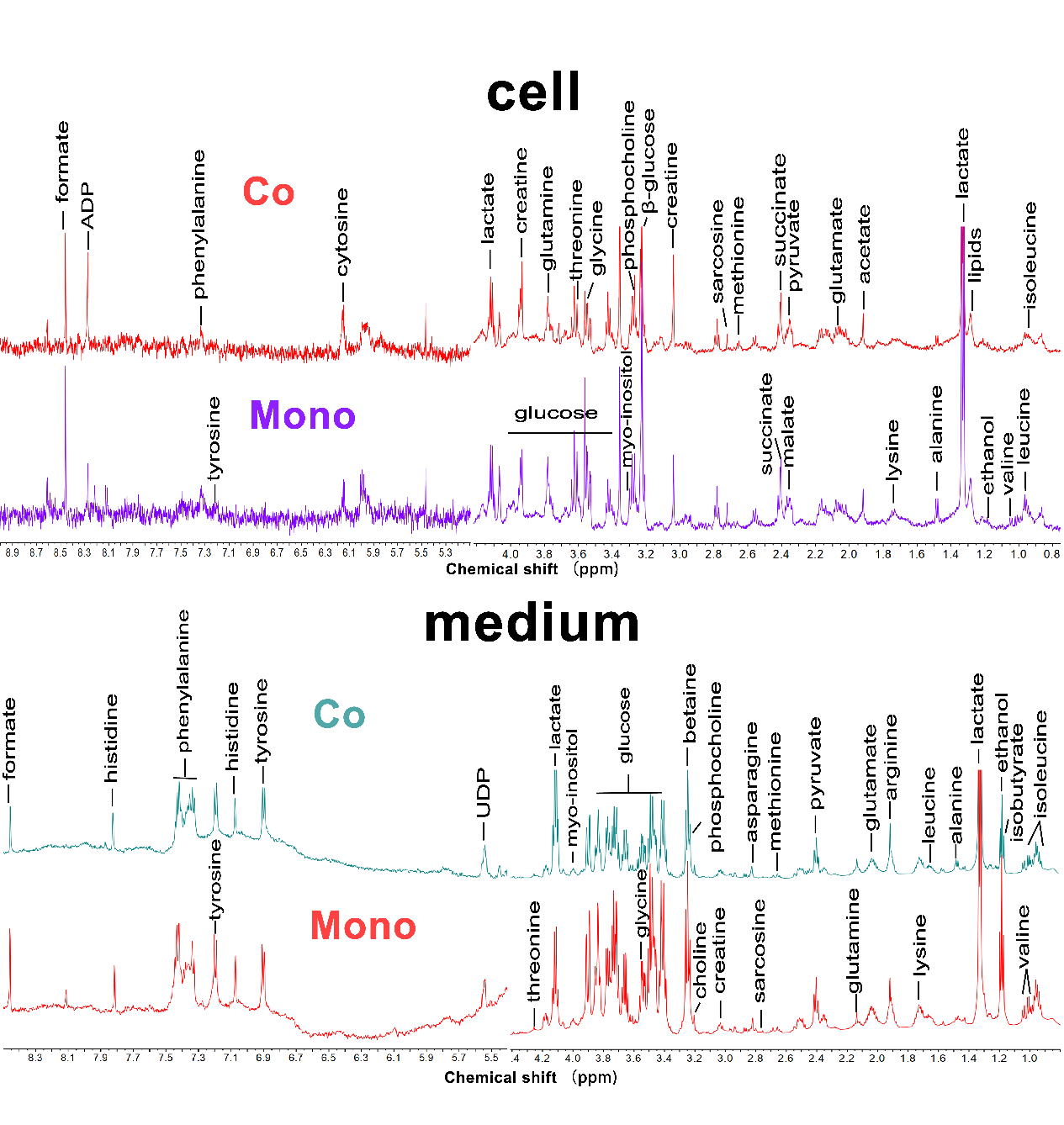


**Figure S5** **^1^H NMR spectra of THP-1 and medium.** Co, co-culture group; Mono, monoculture group.


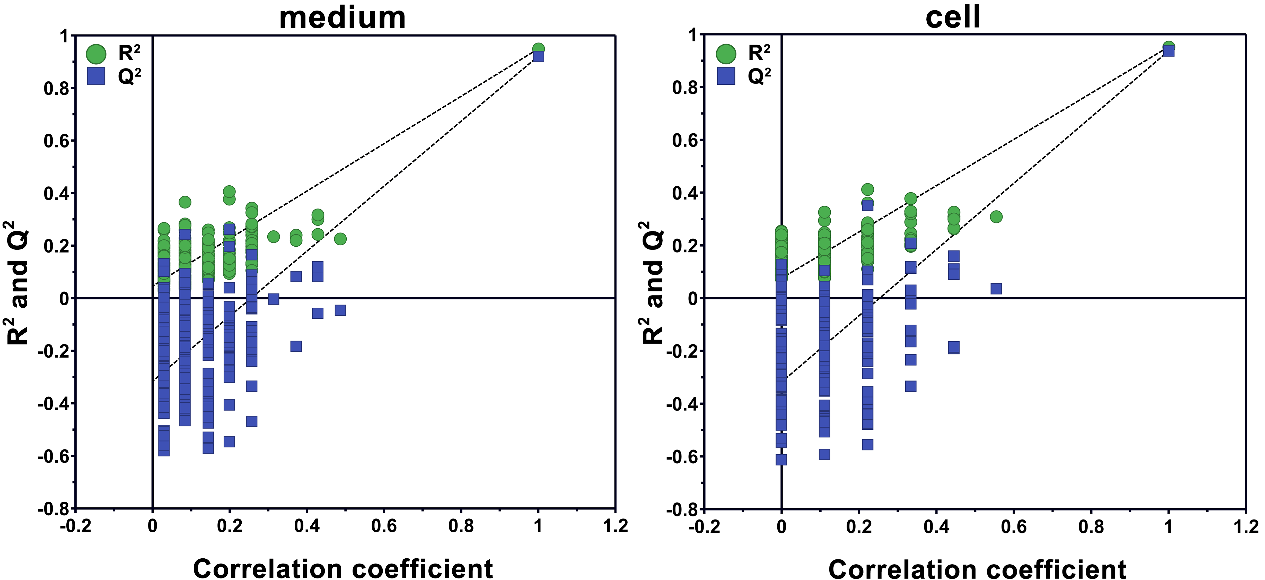


**Figure S6** **The cross-validation result of OPLS-DA depict the discrimination between co-culture and monoculture group in medium and THP-1.**

**
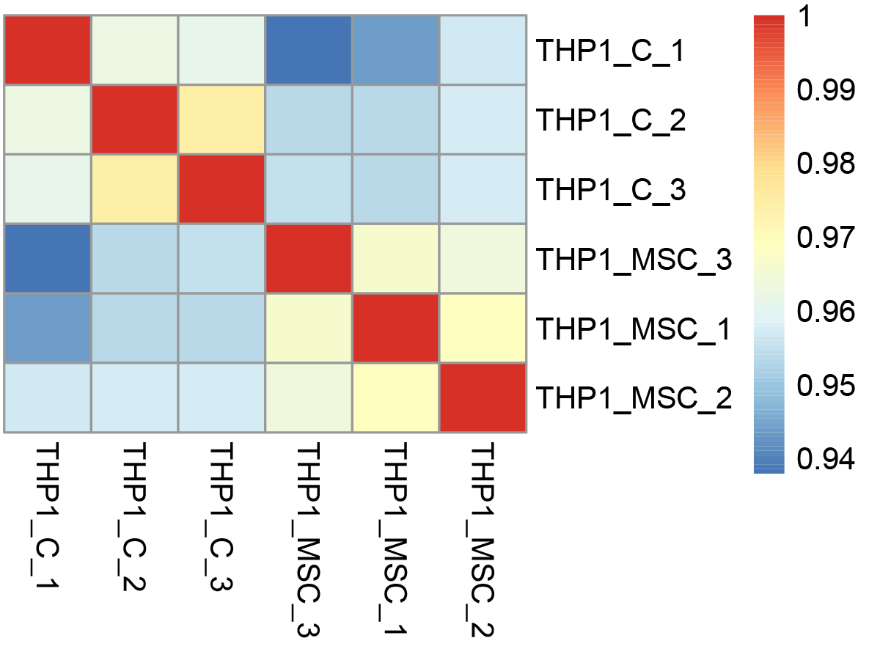
**

**Figure S7** **Heatmap of correlations between transcriptome profiles from co-culture and monoculture THP-1.**

**
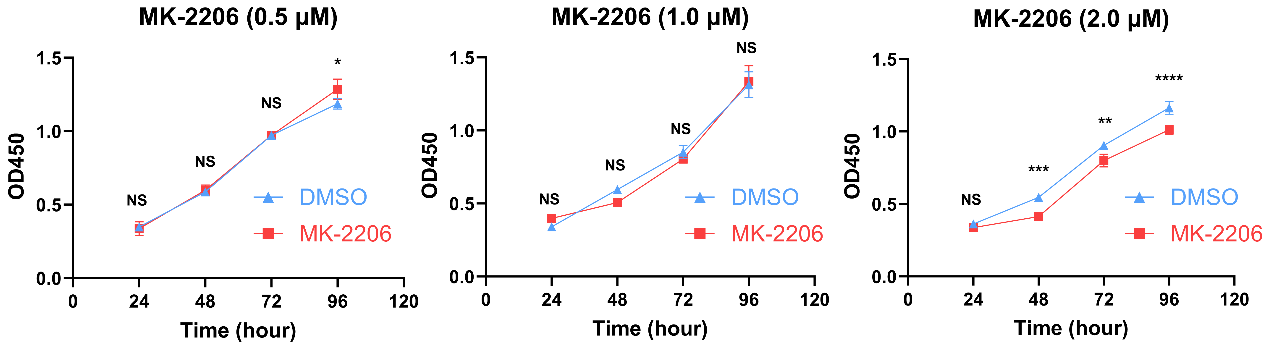
**

**Figure S8** **Cell viability of THP-1 treated with inhibitor alone assessed by CCK-8 assay.** NS, not significant, **p*<0.05, ***p*<0.01, ****p*<0.001, *****p*<0.0001, determined by ANOVA.


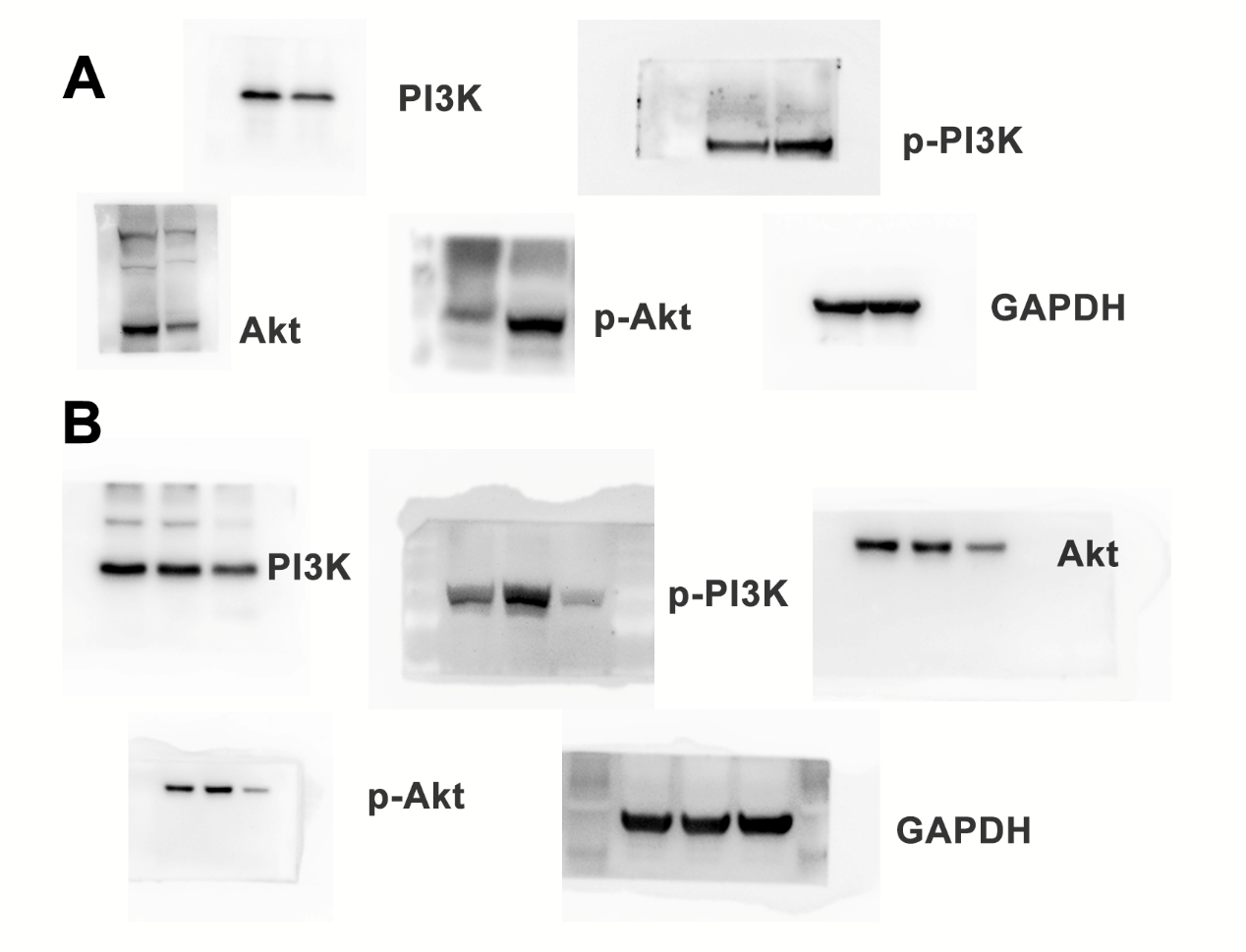


**Figure S9** **Uncropped full-length blots of WB. (A)** Key mediator’s expression of PI3K/Akt signaling pathway in two groups (monoculture and co-culture) (Figure 6A in main file). **(B)** Key mediator’s expression of PI3K/Akt signaling pathway in three groups (monoculture, co-culture, and addition inhibitor in co-culture) (Figure 6B in main file).


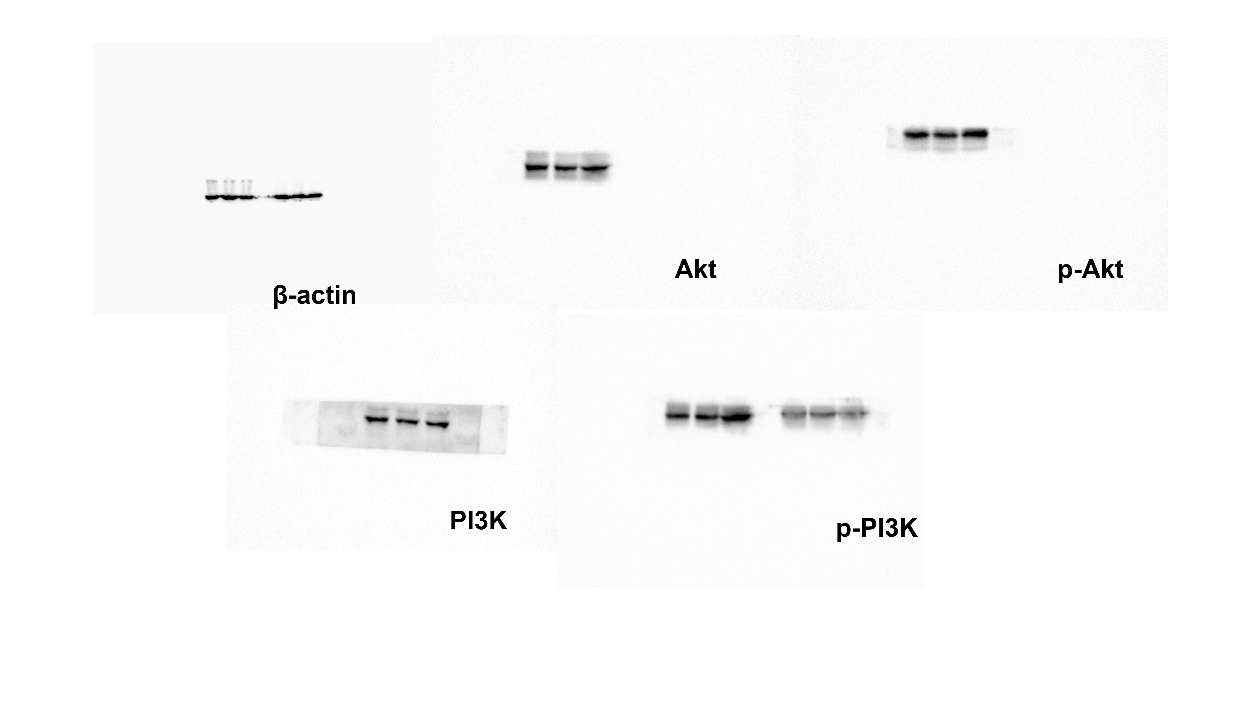


**Figure S10** **Uncropped full-length blots of WB.** Key mediator’s expression of PI3K/Akt signaling pathway in THP-1 cells after IL-6 treatment.

**Table S1.** **Assignments and splitting patterns for metabolites identified by ^1^H NMR analysis of THP-1.**

| **Metabolites** | **^1^H shift (ppm)** |
| --- | --- |
| lipids | 1.22(m^a^), 1.28(br) |
| isoleucine | 0.94(t), 1.02(d) |
| leucine | 0.96(t) |
| valine | 1.01(d), 1.05(d) |
| ethanol | 1.18(t),3.67(t) |
| lactate | 1.33(d), 4.12(q) |
| alanine | 1.49(d), 2.55(t) |
| lysine | 1.74(m), 3.77(t) |
| acetate | 1.92(s) |
| glutamate | 2.05(m), 2.10(m), 2.35(m) |
| glutamine | 2.15(m),2.44(m), 3.78(t) |
| malate | 2.34 (dd) |
| pyruvate | 2.37(s) |
| succinate | 2.42(s) |
| glutathione | 2.95(m), 3.73(t) |
| methionine | 2.65(t) |
| creatine | 3.04(s), 3.93(s) |
| β-glucose | 3.23(dd), 3.43(t), 3.49(t), 3.91(dd), 4.66(d) |
| glycerophosphocholine | 3.22 (s) |
| phosphocholine | 3.24 (s) |
| *myo*-inositol | 3.28(s), 3.62(t), 4.07(t) |
| glycine | 3.56(s) |
| α-glucose | 3.53(dd), 3.84(m), 5.24(d) |
| threonine | 3.59(d) |
| cytosine | 6.01(d) |
| tyrosine | 7.21(d) |
| phenylalanine | 7.32(m), 7.49(m) |
| ADP | 8.27(s) |
| formate | 8.46(s) |

^a^ t, triplet; d, doublet; m, multiplet; s, singlet; q, quartet; dd, doublet of doublets; br, broad.

**Table S2. Assignments and splitting patterns for metabolites identified by ^1^H NMR analysis of medium.**

| **Metabolites** | **^1^H shift (ppm)** |
| --- | --- |
| isoleucine | 0.94(t), 1.02(d), 1.27(d), 1.44(m) |
| leucine | 0.96(t), 1.67(m) |
| valine | 1.00(d), 1.05(d), 2.34(m), 3.62(d) |
| isobutyrate | 1.13(d) |
| unknow | 1.44(s) |
| ethanol | 1.18(t), 3.66(q) |
| lactate | 1.34(d), 4.12(q) |
| alanine | 1.49(d) |
| unknow | 1.50(s) |
| arginine | 1.91(m) |
| lysine | 1.47(m), 1.73(m), 3.03(t), 3.77(t) |
| acetate | 1.92(s) |
| glutamate | 2.04(m), 2.35(m) |
| glutamine | 2.13(m), 2.45(m) |
| proline | 2.39(t) |
| succinate | 2.41(s) |
| methionine | 2.64(t) |
| sarcosine | 2.75(s) |
| creatine | 3.04(s), 3.93(s) |
| asparagine | 2.87(d) |
| betaine | 3.30(s) |
| dimethylamine | 2.72(s) |
| pyroglutamate | 2.51(m), 4.18(t) |
| choline | 3.20(s) |
| phosphocholine | 3.24(s), 3.62(s) |
| β-glucose | 3.36(s), 3.41(m), 3.47(m), 3.49(t), 3.90(dd), 4.66(dd) |
| *myo*-inositol | 4.07(t) |
| glycine | 3.56(s) |
| α-glucose | 3.53(dd), 3.71(t), 3.73(m), 3.84(m), 5.24(d) |
| unknow | 4.17(t) |
| threonine | 4.26(m) |
| unknow | 5.30(s) |
| UDP | 5.56(dd) |
| histidine | 7.08(s), 7.82(s) |
| tyrosine | 6.91(d), 7.20(d) |
| phenylalanine | 7.33(d), 7.37(m), 7.43(t) |
| formate | 8.46(s) |

^a^ t, triplet; d, doublet; m, multiplet; s, singlet; q, quartet; dd, doublet of doublets.
